# Building a modular and multi-cellular virtual twin of the synovial joint in Rheumatoid Arthritis

**DOI:** 10.1038/s41746-024-01396-y

**Published:** 2024-12-24

**Authors:** Naouel Zerrouk, Franck Augé, Anna Niarakis

**Affiliations:** 1 GenHotel, Laboratoire Européen de Recherche Pour La Polyarthrite Rhumatoïde, University Paris-Saclay, University Evry, Evry, France; 2 Sanofi R&D Data and Data Science, Artificial Intelligence & Deep Analytics, Omics Data Science, Chilly-Mazarin, France; 3 Lifeware Group, Inria Saclay, Palaiseau, France; 4 Present Address: University of Toulouse III-Paul Sabatier, Laboratory of Molecular, Cellular and Developmental Biology (MCD), Center of Integrative Biology (CBI), Toulouse, France

**Keywords:** Computational models, Rheumatoid arthritis

## Abstract

Rheumatoid arthritis is a complex disease marked by joint pain, stiffness, swelling, and chronic synovitis, arising from the dysregulated interaction between synoviocytes and immune cells. Its unclear etiology makes finding a cure challenging. The concept of digital twins, used in engineering, can be applied to healthcare to improve diagnosis and treatment for complex diseases like rheumatoid arthritis. In this work, we pave the path towards a digital twin of the arthritic joint by building a large, modular biochemical reaction map of intra- and intercellular interactions. This network, featuring over 1000 biomolecules, is then converted to one of the largest executable Boolean models for biological systems to date. Validated through existing knowledge and gene expression data, our model is used to explore current treatments and identify new therapeutic targets for rheumatoid arthritis.

## Introduction

Rheumatoid arthritis (RA) is a chronic inflammatory autoimmune disease whose causal mechanisms are still not fully understood. RA pathogenesis involves genetic, epigenetic, and environmental factors. Deregulated activation of multiple pathways leads to cartilage degradation and chronic inflammation of the synovial tissue^1^. The inflammatory cascade leads to joint hyperplasia, cartilage damage, and bone destruction. This pathogenic behaviour cannot be associated with a single cell type and results from cellular communication between resident cells and cells from the innate and adaptive immune system. Cell-cell communication determines numerous aspects of the disease’s pathophysiology and can activate or downregulate specific synovial cell populations. It also regulates inflammation, autoimmunity, and articular destruction in the joints by initiating cascades of signalling pathways, further resulting in the expression of proinflammatory molecules and matrix remodelling enzymes. Such cascades trigger disease phenotypes like angiogenesis, cartilage matrix degradation, inflammation, and synovial hyperplasia^2^.

Due to this complexity, there is currently no cure for RA. The proposed treatments seek to relieve disease symptoms and improve survival^3^. However, these therapies have been associated with several adverse effects, and a substantial proportion of RA patients are non-responders^4,5^. This results in patient suffering and increased healthcare costs. Therefore, a better understanding of cellular communication and the intracellular cascades involved in the disease pathogenesis could help elucidate the mode of action (MoA) of current RA drugs and identify new therapeutic options.

Extensive efforts have been undertaken to generate comprehensive datasets to deepen our understanding of the molecular and clinical complexities of RA^6^. The RA-MAP Consortium, for instance, aims to enhance disease management by investigating clinical and biological predictors of treatment response through extensive multi-omics phenotyping^7^. Jiang et al. and Yim et al., on the other hand, integrate genomics with transcriptomics and chromatin accessibility features of RA synovium to characterize the genetic regulation landscape on gene expression and the regulatory mechanisms mediating predisposition to arthritic diseases^8,9^. Additionally, Tsuchiya et al.^10^ performed integrative analyses of mRNA expression, histone modifications, three-dimensional genome architecture and genetic variations that yielded potential therapeutic targets associated with genetic risk of RA. Using clinical data combined with radiographic, ultrasound and serological factors, researchers were also able to identify predictors of RA flares after reaching persistent remission^11,12^.

To effectively manage and interpret the vast amounts of data generated, we need efficient approaches to ingest this knowledge and tailor it to individual patients. An engineering concept known as “digital twins” has been used to analyse and improve complex but deterministic systems like cities and aeroplanes. The goal is to computationally simulate those systems so that they may be developed and tested more rapidly and affordably than in a real-world environment. The digital twin concept can be translated to patients in order to take a step towards personalised healthcare and improve diagnostics and treatment of complex diseases such as RA^13^. This analogy in medicine has gained several successful applications in the field^14^. However, because biological systems are not deterministic by nature and there is no gold standard in the field, their development is still relatively new and presents significant challenges. One possible approach for tackling a task of this magnitude is to transform our system of interest into a deterministic model by (1) building a network model of all the molecular, phenotypic, and environmental aspects that are pertinent to the disease mechanisms, (2) validating its behaviour, and (3) personalising the network in order to consider altered interactions that differ between patients with the same diagnosis. These personalised networks could be treated computationally with thousands of different medications to find the best one and treat the patient with this medication. They could predict disease trajectories, allowing diagnosis before the onset of severe symptoms. They could be used to optimise the timing of suggested medical care and to investigate the effects of potential treatments in a patient-tailored manner. They could also help identify biomarkers or elucidate drug mechanisms of action^15^.

A first step toward building an RA digital twin has been made by developing a mechanistic blueprint for RA, the RA Atlas. It recapitulates existing knowledge related to the intracellular interactions involved in the disease’s pathogenesis in a cell-type and disease-specific manner. The RA Atlas includes, at the time we developed this methodology, four molecular interaction maps specific to the synovial macrophages (including the M1 and M2 phenotypes), synovial fibroblasts and CD4 + T helper 1 (Th1) subtype^16^. However, these cell populations are isolated in the Atlas and do not communicate with each other. Furthermore, these maps are static and cannot be used to generate hypotheses or predictions regarding the system’s behaviour under different perturbations.

Computational modelling is a powerful tool for understanding the emergent features and behaviours of the complex biological systems described in disease maps. Boolean formalism is the most suitable among the modelling techniques available for large-scale biological systems. Indeed, Boolean modelling does not include kinetic parameters that can be difficult to determine in most systems but can use literature and high-throughput technologies to retrieve qualitative data on individual components and interactions^17,18^. However, building and analysing large-scale Boolean models is challenging and computationally demanding^19^. In this direction, an efficient computational framework has been published by N. Zerrouk et al.^20^ to build, analyse and validate the behaviours of large-scale Boolean models. This framework uses molecular interaction maps as a starting point to automatically infer their corresponding executable Boolean models via the CaSQ tool^21^. The generated Boolean models are analysed in a synchronous scheme using a new BMA tool^22^ version deployed to a high-performance computing cluster. The framework identifies all the existing attractors of the models using parallel computing and then tests their coherence against gene expression datasets and prior knowledge. The framework has successfully been applied to generate and validate the RA M1 and M2 macrophage models using their corresponding maps in the RA-Atlas^16^.

**Fig. 1:**
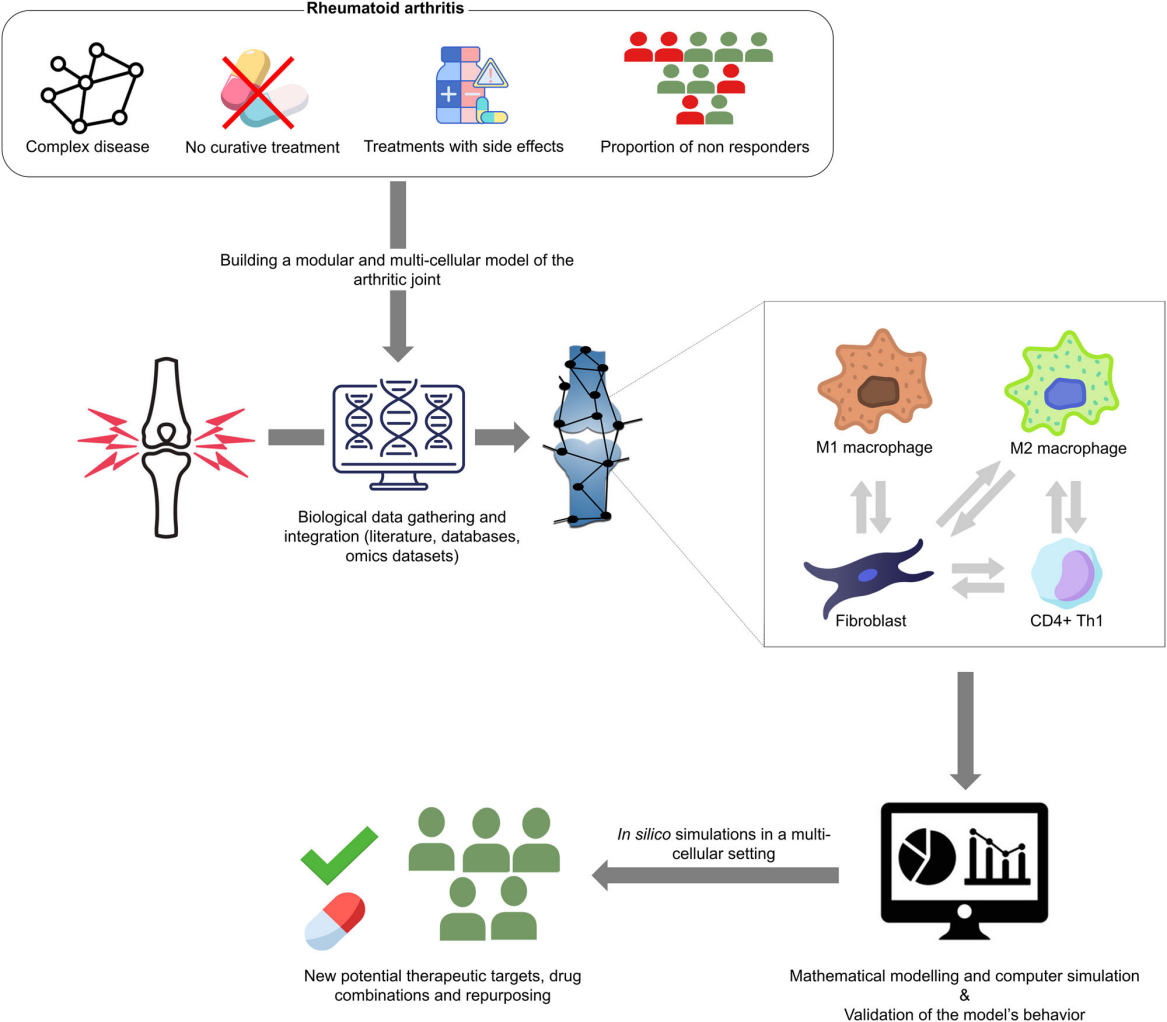
Building a modular multi-cellular virtual twin of the arthritic joint. RA is a complex disease with no curative treatment. The proposed therapies help mitigate inflammation, alleviate pain, and decrease disability associated with RA. Furthermore, these therapies have been associated with several adverse effects, and a substantial proportion of RA patients are non-responders. In this regard, we propose to translate the digital twin concept to RA patients through the construction of a modular multi-cellular model of the arthritic joint. The model is built and validated using literature, database mining, and omics data, and includes four cell-types namely, the M1 and M2 macrophages, the fibroblast, and the CD4 + Th1. The resulting model is used to perform in silico simulations and to identify new therapeutic intervention points and drug combinations for RA.

This work presents our efforts to create a modular, multi-cellular virtual twin of the arthritic joint, as described in Fig. 1. First, we build a multi-cellular map by connecting the RA cell-specific maps present in the RA Atlas with bidirectional intercellular interactions identified using literature, database mining, and omics data. Then, we use the resulting multi-cellular map as an entry point to the computational framework described in ref. ^20^ for the high-throughput combinatorial analyses of large-scale Boolean models. We validate the RA multi-cellular model using prior knowledge and disease- and cell-specific transcriptomic data. The validated model is then used to shed light on the MoA of current RA treatments and to identify new therapeutic intervention points and drug combinations in RA via *single- and double-knockout* in silico simulations.

## Results

### Construction of the RA multi-cellular map

**Fig. 2:**
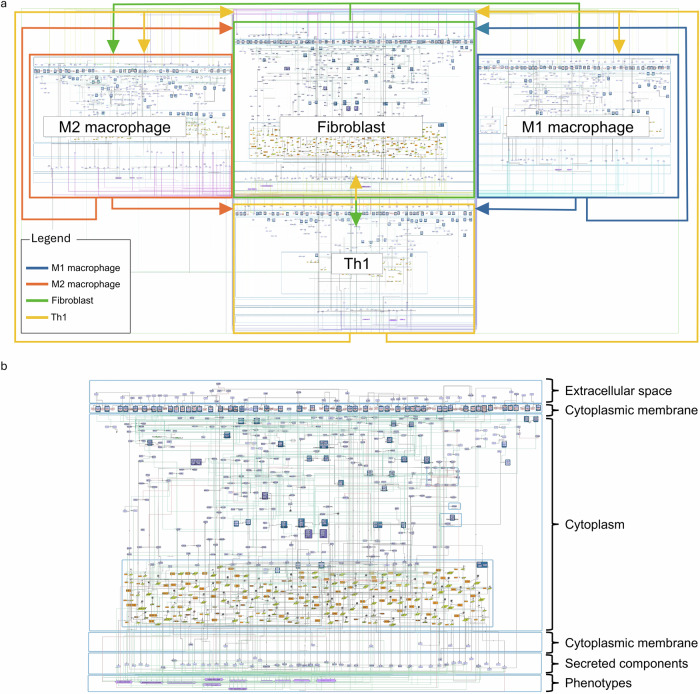
The RA multi-cellular map in CellDesigner. **a** The RA multi-cellular map consists of four cell-specific maps: M1 macrophage (blue square), fibroblast (green square), M2 macrophage (orange square), and Th1 cell (yellow square). Arrows represent bidirectional intercellular interactions, colour-coded according to the source cell type. **b** A close-up view of the fibroblast cell-specific map shows cellular compartments that illustrate signal transduction from the extracellular space to phenotype regulation.

The RA multi-cellular map includes the four cell-specific molecular interaction maps of the RA-Atlas connected via 118 intercellular interactions (Fig. 2). The multi-cellular map comprises 2232 components that interact with one another via 1461 reactions.

Supplementary Tables 1–3 summarise the filtered bidirectional intercellular interactions that connect the RA macrophage, the RA fibroblast, and the RA Th1 cells. Each interaction is associated with diverse resources used to identify it: published literature in PubMed, the internal database of CellPhoneDB and the various pairs of omics datasets used to infer it.

### Generation of the RA cell-specific Boolean models

The generation and calibration of the RA M1 and M2 macrophage models are described in N. Zerrouk et al., 2024^20^. The resulting calibrated states of these models are used in the following analysis.

This section applies the same methodology to build cell-specific Boolean models describing the RA fibroblast and the RA Th1. We used CaSQ^21^ to convert the updated RA cell-specific maps to Boolean models. We first focused on regulating the cell-specific phenotypes represented in these models. Table 1 describes the cell-specific phenotypes for each model and the number of nodes and inputs upstream of these phenotypes. The nodes not involved in regulating the phenotypes of interest were not considered, and the inputs regulating these nodes were fixed at one, the default value in BMA^22^.

**Table 1:** Overview of the RA cell-specific Boolean models

RA cell-specific model	Cell-specific phenotypes	Number of nodes	Number of inputs	Number of interactions
RA fibroblast	Apoptosis, Proliferation, Migration	275	73	446
RA CD4 + Th1	Apoptosis, Proliferation, Migration	120	28	155

The first column indicates the cell-type of interest. The second column displays the associated cell-specific phenotypes for each model. The third, fourth and fifth columns indicate the number nodes, inputs and interactions that are upstream of the cell-specific phenotypes, respectively.

### Computation of all the possible attractors of the RA cell-specific models

Given the high number of inputs in the models, we reduced the list of input combinations by fixing the values of the differentially expressed ones. Overexpressed inputs in RA were fixed at one, and under-expressed inputs in RA were fixed at zero. Based on the information in Supplementary Table 4, 53 out of 73 input states were fixed in the RA fibroblast model. The number of input combinations was then equal to 2^20^. We used the BMA tool deployed to a machine with 96 single-core CPUs and 768 GB of RAM to run the attractors’ search. All the resulting attractors were steady states and were kept for further analysis.

Regarding the CD4 + Th1 model, 16 out of 28 inputs were fixed according to the information displayed in Supplementary Table 5. The number of input combinations was then equal to 2^12^. All the corresponding attractors were steady states.

### Validation of the cell-specific models’ behaviour

First, we filtered the steady states according to the values of their cell-specific phenotypes. These phenotypes’ biologically coherent Boolean values were extracted from the literature in disease- and cell-specific manners. In the RA fibroblast model, the state of the cell-specific phenotypes should reflect their resistance to apoptosis and excessive proliferation in RA synovium (apoptosis phenotype should be OFF, and proliferation phenotype should be ON in the model)^23^. They also describe the fibroblast’s ability to migrate to adjacent joint structures contributing to cartilage destruction^24–26^ (migration phenotype should be ON in the model). All RA fibroblast model’s steady states passed through this filtering step.

In the RA CD4 + Th1 model, the state of the cell-specific phenotypes should describe the increased ability of this cell type to migrate and extravasate from blood vessels to the inflamed RA joint (migration phenotype should be ON in the model)^27^. They also show their increased apoptosis resistance (apoptosis phenotype should be OFF in the model) and their hyperactivation and impaired proliferation in RA (proliferation phenotype should be ON in the model)^28^. One thousand twenty-four steady states passed through this filtering step in the RA CD4 + Th1 model.

For each cell-specific model, we identified their differentially expressed molecules (see Methods section) and calculated similarity scores between these lists of differentially expressed molecules (Supplementary Tables 4 and 5) and their matching nodes in each filtered steady state. Steady states with the highest similarity score in each model were selected, and their average vectors were calculated. The resulting mean vectors represent the calibrated state of the RA cell-specific models.

Regarding the RA fibroblast model, 4096 steady states had the highest similarity score, and their average vector was calculated. In the resulting vector, 254 nodes were fixed at zero or one, while 21 were not fixed (Supplementary Table 6). This model’s state can reproduce 99% of the experimentally observed discretized values.

Regarding the RA Th1 model, 128 steady states had the highest similarity score. In their resulting mean vector, 109 out of 120 nodes were fixed at zero or one. Eleven nodes were not fixed (Supplementary Table 7). This model’s state can reproduce 100% of the observed discretized values.

### Generation and calibration of the RA multi-cellular model

First, we used CaSQ to convert the RA multi-cellular interaction map to a Boolean model. It consists of 1104 nodes, including 240 inputs and 1845 interactions. To calibrate the resulting multi-cellular model, we first combined the cell-specific models’ calibrated states (including the M1 and M2 macrophages’ states that we obtained from N. Zerrouk et al., 2024^20^) via the addition of intercellular interaction present in the multi-cellular model. Adding these interactions allowed us to fix additional nodes in the resulting multi-cellular model. Indeed, among the 240 inputs present in the multi-cellular model, 141 were already fixed in the RA cell-specific models’ calibration and combination. We used information from literature and gene expression datasets to fix more inputs. Based on the information in Supplementary Table 8, 78 additional inputs were fixed. The total number of fixed inputs was equal to 219. The 21 remaining inputs were not associated with a permanent Boolean value. The total number of input combinations was then equal to 2^21^. We used the BMA tool deployed to a machine with 96 single-core CPUs and 768 GB of RAM to run the attractors’ search. All the resulting attractors were steady states and were kept for further analysis.

We calculated the similarity score between the list of differentially expressed molecules (Supplementary Table 8) and their matching nodes in each steady state. Steady states with the highest similarity score were selected to calculate their mean vectors. The resulting mean vector represents the calibrated state of the RA multi-cellular model. Thirty-two thousand seven hundred sixty-eight steady states had the highest similarity score. In their mean vector, 1076 out of 1104 nodes were fixed at zero or one, while 28 of them were not fixed (Supplementary Table 9). This multi-cellular model’s state can reproduce 98,8% of the observed Boolean values used to calibrate it.

### Testing the effects of therapeutic targets’ single knockouts on the RA multi-cellular model

To identify potential therapeutic targets in the RA multi-cellular model, we perform an exhaustive search using the Therapeutic Target Database (TTD)^29^. We only focus on inhibiting the drug target to simulate knockouts in the model. We screen the targets based on the Mode Of Action (MOA) of their associated drugs and only keep the ones that can be targeted by at least one inhibitor (1643 targets).

Among the 1643 potential therapeutic targets present in TTD, 194 were identified in our RA multi-cellular model (Supplementary Table 10). We mimic the effect of these 194 potential therapeutic targets using in silico knockout simulations. We use the calibrated state of the RA multi-cellular model as initial simulation conditions (Supplementary Table 9). The models’ phenotype states after the target knockouts are compared to their corresponding calibrated states. Table 2 summarises the identified therapeutic targets and their effects on the model’s phenotypes, while Table 3 describes the identified targets.

**Table 2:** Therapeutic targets from the TTD database that perturb RA phenotypes in the RA multi-cellular model

Successful targets	Phenotypes of the RA multi-cellular model														
	M1 macrophage			M2 macrophage		Fibroblast			Th1			Synovial joint			
	Apoptosis	Proliferation	Osteoclastogenesis	Apoptosis	Proliferation	Apoptosis	Proliferation	Migration	Apoptosis	Proliferation	Migration	Inflammation	Angiogenesis	Matrix degradation	Osteoclastogenesis
AKT2							↘	↘							
CAV1						↗	↘	↘							
CREB1							↘								
GSK3β				↘	↗										
ERK1		↘	↘												
MIR221													↘		
mTOR									↗	↘	↘				
NF-κB	↗	↘	↘					↘		↘	↘			↘	
TBX21									↗	↘	↘				

The first four columns correspond to the cell-specific phenotypes present in each of the cell-specific models. The fifth column represents the phenotypes describing the overall joint’s condition. The downward arrows describe the inhibition of the active phenotypes in the calibrated state of the model. Upward arrows describe the activation of the phenotypes that were inhibited in the calibrated state of the model. The absence of arrows means that the phenotype state remains unchanged.

**Table 3:** Description of the therapeutic targets perturbing the RA phenotypes in the RA multi-cellular model

Successful targets	Target type	Associated disease(s)	Drugs with the highest status
CAV1	Literature-reported target	/	/
AKT2	Literature-reported target	/	Akt inhibitor VIII (Investigative)
CREB1	Literature-reported target	/	/
NF-κB	Successful target	Irritable bowel syndrome, Rheumatoid arthritis, Choreiform disorder, Lupus erythematosus, Multiple sclerosis…	Sulfasalazine (Approved)
TBX21	Literature-reported target	/	/
MTOR	Successful target	Arteries/arterioles disorder, Chronic myelomonocytic leukaemia, Hydrocephalus, Multiple myeloma, Renal cell carcinoma	Everolimus (Approved)
ERK1	Clinical trial target	Melanoma, Pancreatic cancer, Cancer, Arteries/arterioles disorder, Mature T-cell lymphoma	BVD-523 (Phase 2)
GSK3β	Clinical trial target	Myotonic disorder, Acute myeloid leukaemia, Osteosarcoma, Fragile X chromosome, Myeloproliferative neoplasm	Tideglusib (Phase 2/3)
MIR221	Literature-reported target	/	/

The target types are based on the TTD database’s categories and go from successful targets to literature-reported targets. The drugs displayed in this table were also extracted from the TTD database and were selected based on their highest status.

**Fig. 3:**
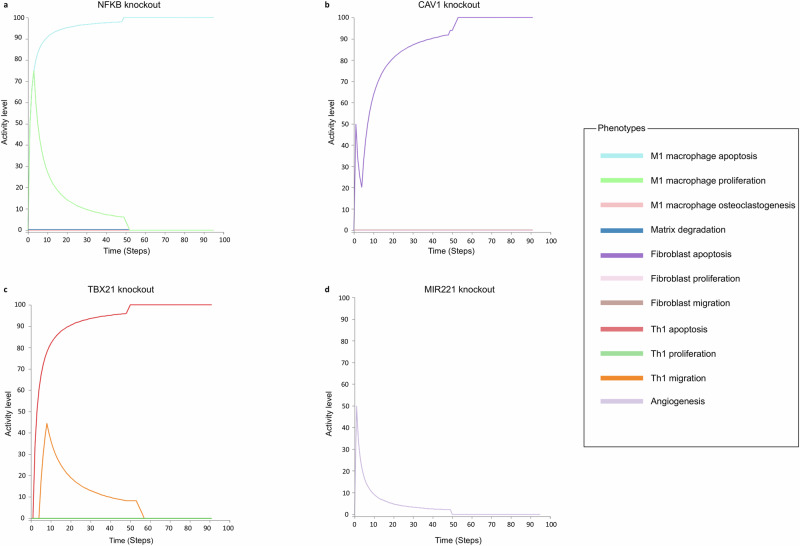
Single knockout simulations of the therapeutic targets that perturb the multi-cellular model’s phenotypes. Simulations were performed using the Cell Collective platform^30^. Each point in the graphs represents the activity level of the displayed node. It is calculated as the number of logical time steps in which the displayed node is active over a user-defined number of time steps. **a** NF-κB knockout in the calibrated RA model induces the M1 macrophage’s apoptosis, inhibits the M1 macrophage’s growth and differentiation into osteoclasts, and suppresses the degradation of the extracellular matrix. **b** CAV1 knockout in the calibrated RA model induces the apoptosis of the RA fibroblasts while suppressing their proliferation and migration. **c** TBX21 knockout in the calibrated RA model leads to the suppression of Th1 proliferation and migration and to the activation of the Th1 apoptosis. **d** MIR221 knockout in the calibrated RA model leads to the inhibition of the angiogenesis phenotype.

**Fig. 4:**
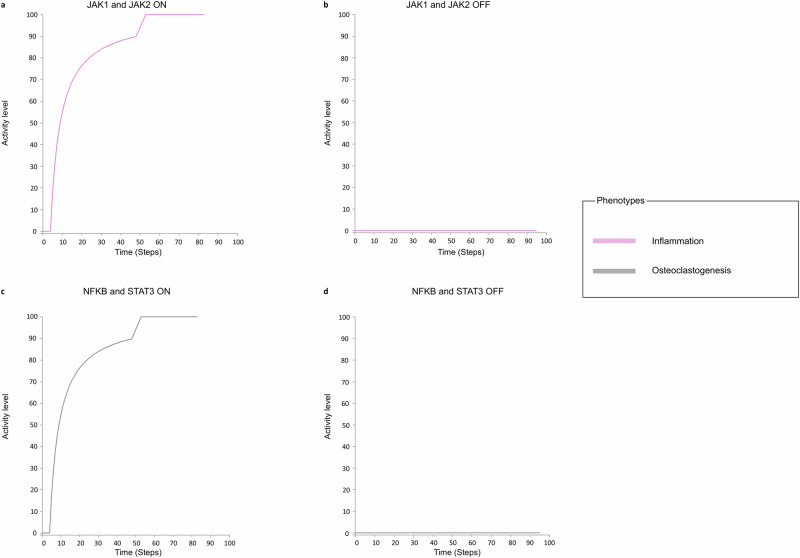
Double knockout simulations of the drug target combinations that perturb the multi-cellular model’s phenotypes. Simulations were performed using the Cell Collective platform^30^. Each point in the graphs represents the activity level of the displayed node. It is calculated as the number of logical time steps in which the displayed node is active over a user-defined number of time steps. **a** Simulation with JAK1 and JAK2 active in the model. The Inflammation phenotype is active. **b** Simulation with JAK1 and JAK2 inactive in the model. The inflammation phenotype gets inhibited. **c** Simulation with NF-κB and STAT3 active in the model. The Osteoclastogenesis phenotype is active. **d** Simulation with NF-κB and STAT3 inactive in the model. The Osteoclastogenesis phenotype gets inhibited.

**Fig. 5:**
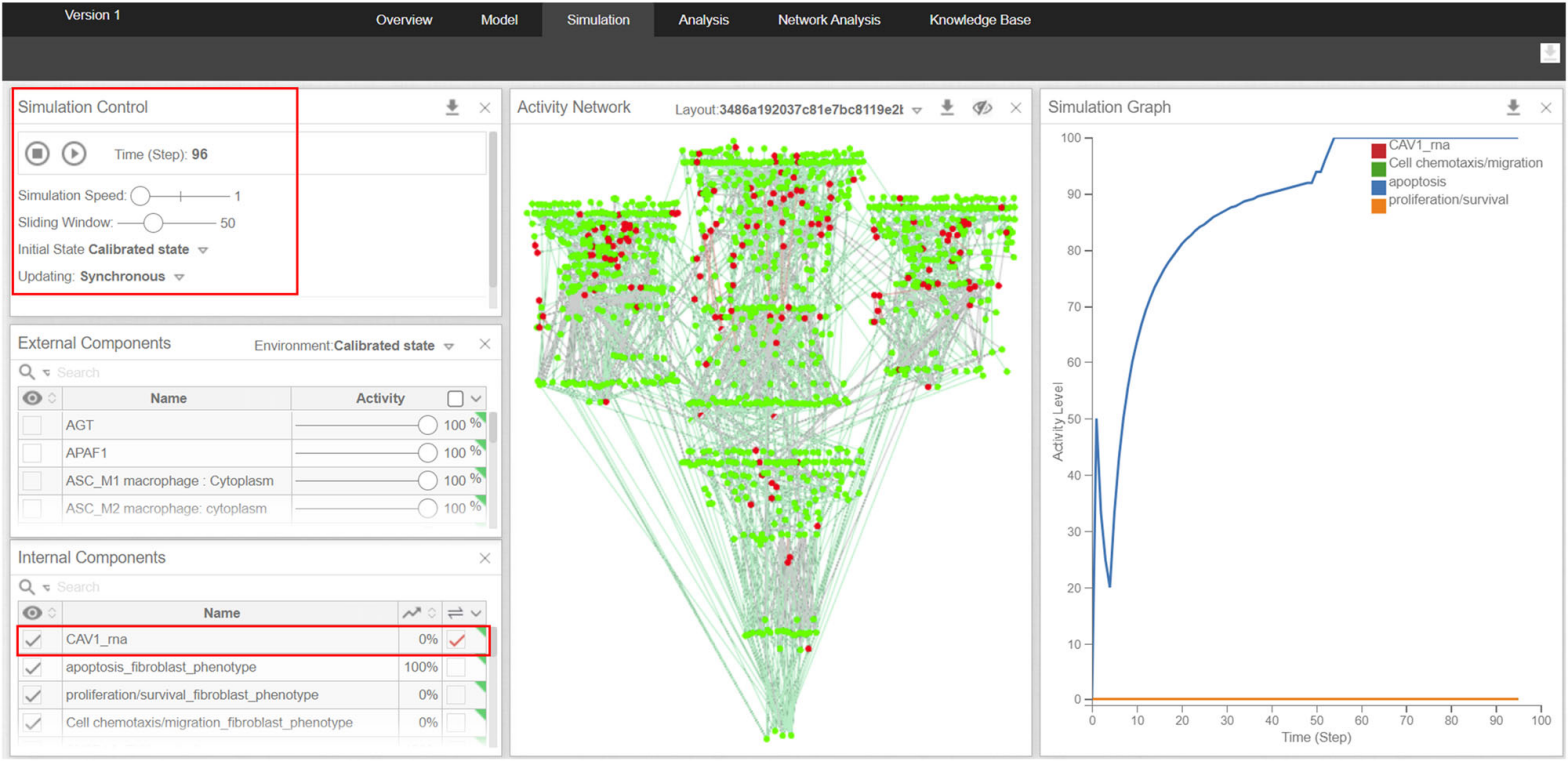
Example of the In silico simulation workflow of the RA multi-cellular model using the Simulation tool of the Cell Collective platform^30^. The Simulation Control panel indicates the parameters used to conduct the experiment with the initial state being the calibrated state of the model. The External Components panel shows the input nodes present in the model. Their state is set according to their Boolean values in the calibrated state of the model (0% for zero, 100% for one). The Internal Components panel indicates the nodes that are not inputs in the model. The figure illustrates the in silico KO of CAV1 which is set as mutated with loss of function. The Activity Network panel in the middle shows the changes in the states of the model’s nodes throughout the experiment. Red nodes indicate inactivity, while green nodes indicate activity. The Simulation Graph illustrates the activity level of the displayed nodes.

To visualize the simulation results, we imported our model in an SBML qual format in the Cell Collective platform^30,31^. The platform allows the simulation of loss/gain of functions of user-defined nodes. As we can see it in Figs. 3 and 4, each point in the graphs provided by the platform represents the number of logical time steps in which the displayed node is active over a user-defined number of time steps called sliding window (e.g. if the sliding window is 100, and the node is active in 20 steps over the last 100 steps, the activity level displayed at that point on the graph will be 20%). The simulations were conducted with a synchronous update mode, a simulation speed of one, and a sliding window of 50, observing the model’s behaviour over 100 steps (Fig. 5).

N. Zerrouk et al.^20^ showed that GSK3β inhibition induced the M2 macrophage model’s proliferation while suppressing the apoptosis phenotype. GSK3β is involved in the expression of multiple glycolytic genes. These results are in accordance with Alivernini et al., 2020’s findings^32^ regarding the lower expression of glycolytic pathways in macrophage populations from healthy donors and remission RA compared to the ones from active RA.

N. Zerrouk et al.^20^ also showed that NF-κB inhibition induced the M1 macrophage model’s death and inhibited the M1 macrophage model’s growth, and that ERK1 inhibition suppressed the proliferation phenotype in the inflammatory macrophage model.

The simulations we performed on the multi-cellular model reinforce these results by showing that GSK3β KO did not affect the other cell types present in the model, that NF-κB and ERK1 inhibitions suppressed the M1 macrophage differentiation into osteoclasts (Fig. 3a) as well and that NF-κB KO perturbed the behaviour of other cell-type in the synovium (Table 2).

CAV1 KO induced the apoptosis phenotype of the RA fibroblasts while suppressing their proliferation and migration phenotypes (Fig. 3b). AKT2 KO, on the other hand, inhibited their proliferation and migration phenotypes while preserving their ability to resist apoptosis. CREB1 inhibition led to suppressing the RA fibroblast’s proliferation phenotype but did not affect their capacity to migrate to other joints or resist apoptosis. We can also see that fibroblast migration can be suppressed via NF-κB inhibition.

Regarding the Th1 subtype, two targets were identified: MTOR and TBX21. The inhibition of these targets led to the suppression of Th1 excessive proliferation and migration phenotypes and the activation of Th1 apoptosis (Fig. 3c). NF-κB KO, on the other hand, inhibited their proliferation and migration phenotypes while preserving their ability to resist apoptosis.

Potential therapeutic targets that perturb the general biological condition of the RA joint were also identified. MIR221 inhibition led to the suppression of the angiogenesis phenotype in the RA synovium (Fig. 3d), and NF-κB inhibition suppressed the degradation of the extracellular matrix.

### Testing the effects of therapeutic targets’ double knockouts on the RA multi-cellular model

The targets were combined in pairs to investigate the potential synergistic effect of the previously tested therapeutic targets. The RA multi-cellular model was then used to predict the outcome of these double KOs. We used the same initial conditions for the mono drug testing; then, we compared the perturbed states with their corresponding calibrated state.

We generated a list of all the possible pairs (without repetition) of the 194 previously identified therapeutic targets. Eighteen thousand seven hundred twenty-one drug combinations in total were tested.

Three synergistic pairs were identified, namely ERK1/NOTCH1, JAK1/JAK2 and NF-κB /STAT3. Table 4 summarises the identified synergistic pairs and their effects on the model’s phenotypes, while Table 5 describes the identified targets.

**Table 4:** Combinations of therapeutic targets (from the TTD database) that perturb RA phenotypes in the RA multi-cellular model

Successful combination	Targeted phenotypes														
	M1 macrophage			M2 macrophage		Fibroblast			Th1			Synovial joint			
	Apoptosis	Proliferation	Osteoclastogenesis	Apoptosis	Proliferation	Apoptosis	Proliferation	Migration	Apoptosis	Proliferation	Migration	Inflammation	Angiogenesis	Matrix degradation	Osteoclastogenesis
ERK1 and NOTCH1	↗	↘	↘												
JAK1 and JAK2	↗	↘	↘									↘			
NF-κB and STAT3	↗	↘	↘					↘		↘	↘			↘	↘

The first four columns represent the cell-specific phenotypes present in each of the cell-specific models. The fifth column represents the phenotypes describing the overall joint’s condition. Downward arrows describe the inhibition of the active phenotypes in the calibrated state of the model. Upward arrows describe the activation of the phenotypes that were inhibited in the calibrated state of the model. The absence of arrows means that the phenotype state remains unchanged.

**Table 5:** Description of the combinations of therapeutic targets perturbing the disease phenotypes in the RA multi-cellular model

Synergistic combination	Targets	Target type	Associated disease(s)	Drugs with the highest status
JAK1/JAK2	JAK1	Successful target	Acquired hypomelanotic disorder, Atopic eczema, Crohn’s disease, Myeloproliferative neoplasm, Pancreatic cancer, …	Baricitinib (Approved)
	JAK2	Successful target	Acquired hypomelanotic disorder, Atopic eczema, Myeloproliferative neoplasm, Pancreatic cancer, Rheumatoid arthritis, …	Baricitinib (Approved)
ERK1/Notch1	ERK1	Clinical trial target	Melanoma, Pancreatic cancer, Cancer, Arteries/arterioles disorder, Mature T-cell lymphoma	BVD-523 (Phase 2)
	Notch1	Clinical trial target	Lymphoma, Mature T-cell lymphoma, Cancer	LY3039478 (Phase 1/2)
NF-κB/STAT3	NF-κB	Successful target	Irritable bowel syndrome, Rheumatoid arthritis, Choreiform disorder, Lupus erythematosus, Multiple sclerosis, …	Sulfasalazine (Approved)
	STAT3	Successful target	Psoriasis, Brain cancer, Colorectal cancer, Liver cancer, Malignant digestive organ neoplasm.	Acitretin (Approved)

Each combination is composed of individual targets displayed in the Targets column. The target types are based on the TTD database’s categories and go from successful targets to literature-reported targets. The drugs displayed in this table were also extracted from the TTD database and were selected based on their highest status.

N. Zerrouk et al.^20^ already identified ERK1/NOTCH1 and JAK1/JAK2 as promising combinations to reestablish the RA M1/M2 ratio.

The simulations we performed further support these results and show a more substantial impact of these double KOs at the multi-cellular level. Indeed, both ERK1/Notch1 and JAK1/JAK2 KOs lead to the suppression of the M1 macrophage’s osteoclastogenesis, and JAK1/JAK2 double KO inhibits the inflammation in the RA synovium (Fig. 4a, b).

A new pair that acts synergistically in our model was also identified. The combination of NF-κB and STAT3 KOs inhibits the differentiation of the osteoclast precursor cells in the RA synovium (Fig. 4c, d).

## Discussion

Digital twin is an emerging technology that builds on the convergence of computer science, mathematics, and artificial intelligence. Its exponential development is supported by the rapid growth of communication and sensor technologies, extensive data analysis, virtual reality, the Internet of things, and simulation technologies^33^.

Digital twin implementation in healthcare has the potential to advance biomedical research with applications for personalised medicine, pharmaceutical development, and clinical trials^34^. Current tangible implementations of digital twins can be found in precision cardiology^35^, type 1 diabetes^36^, cancer^37^, and epidemic outbreaks^38^. In these applications, researchers combine several cutting-edge technologies, including mathematical modelling.

This work initiates the development of a virtual twin of the arthritic joint, first by constructing a comprehensive large-scale map that depicts both the intra- and intercellular interactions involved in RA pathogenesis. The map incorporates the four cell-specific maps of the RA Atlas, describing the synovial fibroblast, M1 and M2 macrophages, and CD4 + Th1 cell-types. Furthermore, it integrates bidirectional cellular communication between these cell types, providing a detailed multi-cellular representation of the RA synovium. The map is modular, allowing for future expansion with additional cell-specific maps.

To explore the emergent behaviour of the system, we employed the Boolean formalism. Boolean models can handle large-scale systems and do not require quantitative parameters. We used the map to model translation framework and the tool CaSQ described in Aghamiri et al., 2020 to translate the multi-cellular map to a fully executable, large-scale Boolean model^20^. The dynamic behaviour of the RA multi-cellular model was tested against prior knowledge to assess its capacity to reproduce known biological mechanisms. The RA multi-cellular model is significantly larger in scale compared to the two macrophage models tested in Zerrouk et al., 2024, demonstrating the scalability of the proposed computational framework.

The model was then used to study the mechanism of action of current RA treatments and identify new potential therapeutic targets and drug combinations. In silico simulations on the calibrated RA multi-cellular model identified AKT2 as a potential target for inhibiting RA fibroblast proliferation and migration phenotypes. Several studies support the concept of AKT2 inhibition for therapeutic intervention in RA. They showed that blocking the AKT pathway inhibits RA progression^39^. They also demonstrated that in vitro siRNA-mediated down-regulation of AKT2 significantly prevented cell proliferation and migration of human RA fibroblasts^40,41^.

Caveolin 1 (CAV1) was also highlighted as a potential target for inducing apoptosis and inhibiting proliferation and migration in RA fibroblasts. Studies support this prediction by showing that in vitro silencing of CAV1 in RA drastically reduces cell proliferation and promotes apoptosis in human RA fibroblasts. On the other hand, enforced expression of CAV1 in RA fibroblasts restores cell proliferation and attenuates apoptosis^42^. CAV1 was also demonstrated to drive resistance to apoptosis in a large-scale Boolean model describing the RA synovial fibroblasts^43^.

Simulations of CREB1 KO in the model led to inhibiting the proliferation phenotype in the RA fibroblasts while maintaining their capacity to resist apoptosis. This inhibitory effect was demonstrated in an experimental study where in vitro suppression of CREB activity downregulates aberrant synovial cell functions in patients with RA via suppressing the RA synovial fibroblast proliferation^44^. CREB1’s effect on cellular proliferation was highlighted in in silico simulations on a published large-scale Boolean model of RA fibroblasts^43^. These results further highlight AKT2, CREB1, and CAV1 as promising targets for downregulating hyperactive fibroblasts in the rheumatic joint.

Knocking out NF-κB in the multi-cellular model inhibited the differentiation of the M1 macrophages into osteoclasts. It also affected other cell types via the suppression of migration in RA fibroblasts, proliferation, and migration in the RA Th1 subtype, and degradation of the extracellular matrix in the joint. These results are supported by experimental evidence. Indeed, researchers have demonstrated that inhibiting NF-κB signalling blocks the expression of several Matrix MetalloProteinases (MMPs), which are responsible for destroying the extracellular matrix and the articular cartilage in RA^45^. Blockade of MMPs’ expression suppresses RA synovial fibroblast migration and invasion^46^. The critical importance of NF-κB in bone turnover has also been highlighted experimentally. It was shown that inhibition of NF-κB was a practical approach to inhibit osteoclast formation and bone resorptive activity and displayed anti-inflammatory and anti-osteolytic benefits^47^. In addition, several studies have described the role of NF-κB in Th1 differentiation. They showed that Th1 responses were significantly impaired, and IFN-g production was abrogated due to diminished NF-κB activation^48^.

Moreover, the RA multi-cellular model identified two potential therapeutic targets to downregulate the RA CD4 + Th1: MTOR and TBX21 (or T-bet). Studies showed that CD4+ cells from T-bet-/- mice are skewed toward an anti-inflammatory Th2 differentiation via the expression of high levels of GATA-3. This GATA-3’s gain of function after TBX21 depletion was also highlighted in the KO simulations we performed on the RA multi-cellular model. Hence, regulation of the T-bet/GATA-3 ratio can reduce inflammatory damage to RA cells, and the mechanism behind it may be related to regulating the Th1/Th2 ratio of RA cells through T-bet depletion^49^.

MTOR, on the other hand, has also been found to play essential roles in Th1 cell development. It was found that CD4 + T cells deficient in MTOR failed to differentiate into effector Th1 cells^50^. In RA, mTOR inhibition has also shown efficacy in reducing joint inflammation in animal models of arthritis^51^ and in patients with RA^52^.

Our results also showed that JAK1/JAK2 double KO suppressed the chronic inflammation of the RA synovium and the differentiation of macrophages to osteoclasts. Baricitinib, an oral JAK inhibitor selective for JAK1 and JAK2, is approved by the Food and Drug Administration (FDA) for treating RA^53^. It prevents the activation of STAT pathways, decreasing systemic inflammation and the progression of bone destruction associated with RA^54^.

MIR221 was revealed as a potential target to downregulate angiogenesis. Studies support this finding and demonstrate that MIR221 can downregulate THBS1, which acts as an anti-angiogenic factor on endothelial cells^55^. Thus, inhibiting MIR221 would restore THBS1 expression, which, in RA, was found to help restore tissue homoeostasis during resolution of inflammation^56^.

Lastly, the RA multi-cellular model identified a potential new synergistic pair of therapeutic targets via double KOs simulations. NF-κB and STAT3 double KO inhibited the differentiation of osteoclast precursor cells in the RA multi-cellular model. Osteoclastogenesis strictly depends upon support from accessory cells, which supply cytokines required for osteoclast differentiation^57^. CSF1, RANKL, TNF-α and IL-6 have been found to induce the differentiation of osteoclasts and bone resorption activity in RA patients^58^. STAT3 and NF-κB are essential transcription factors for the expression of these osteoclastogenic factors’ expression^59–61^. Therefore, targeting STAT3 and NF-κB could be a promising strategy to inhibit bone erosion in RA.

In this work we select cell types and subpopulations based on (a) data availability, (b) interest of cell-cell communication, (c) feasibility of the simulations in regard to the size of the model. We had to overcome several technical difficulties, scalability issues, computational power, simulation parallelization and face the lack of publicly available and accessible data that could be integrated into this work. Our model is far from complete, but it is a very important milestone, from both a computational and biological/pathological aspect. Indeed, the RA multi-cellular map and model presented in this work are the largest curated representations of the RA synovium to date. Models and networks for RA have been previously proposed but are either large-scale networks inferred using gene expression data^62–64^, and thus more noisy than manually curated ones; or computational models based on differential equations^65,66^. However, these quantitative models are limited in size and cannot be used to describe entire cellular pathways in detail. Recently published large-scale Boolean models for RA challenge the size limitation, however, they remain of smaller scale than the RA multi-cellular model; and only focus on the active role of fibroblasts and macrophages without considering cell-cell communication^20,43,67^.

Building a successful digital twin for RA necessitates a diverse range of data types, each contributing unique insights to model the complexity of the disease. Integration of additional multi-omics data, including genomics, proteomics, and metabolomics, combined with demographic data (sex, age) and clinical data (symptoms, disease progression, treatment responses) would further enhance the model’s accuracy. We also wish to expand the RA multi-cellular map and model with molecular interaction maps of additional cell types such as B cells, endothelial cells, osteoblasts, and osteoclasts and enrich existing cell-specific maps with relevant metabolic pathways. We also aim at refining our models to represent the phenotypic diversity of some of the cell types of interest, especially fibroblasts, but also representing more intermediate states of macrophages. Our model building approach is quite modular and can be expanded with the addition of more cell types and sub-types.

The next steps of this work include personalizing the RA template model for individual patients. Contextualizing the multi-cellular model with patient data would enable the creation of patient-specific digital twins capable of replicating crucial features unique to each individual. Ideally, a digital twin for RA should factor in patient-specific customization parameters, and treat a live feed of data. However, significant gaps remain, particularly in longitudinal multi-omics data that capture individual variability in disease mechanisms and treatment responses. There are also important challenges regarding the feasibility of a fully connected RA digital twin with real-time updating. Addressing these gaps requires collaborative efforts to generate high-resolution, longitudinal datasets and innovative technologies to better characterize and monitor RA joint dynamics, as well as the systemic, pathophysiological manifestations of the disease. Therefore, implementing the RA multi-cellular model in a computational ecosystem designed to process data from sensors and imaging technologies, via AI algorithms, along with physiological measurements, could revolutionize personalized care of RA patients, and enable clinicians to test hundreds of different scenarios in a short amount of time.

## Methods

### General workflow for constructing and calibrating the RA multi-cellular model

**Fig. 6:**
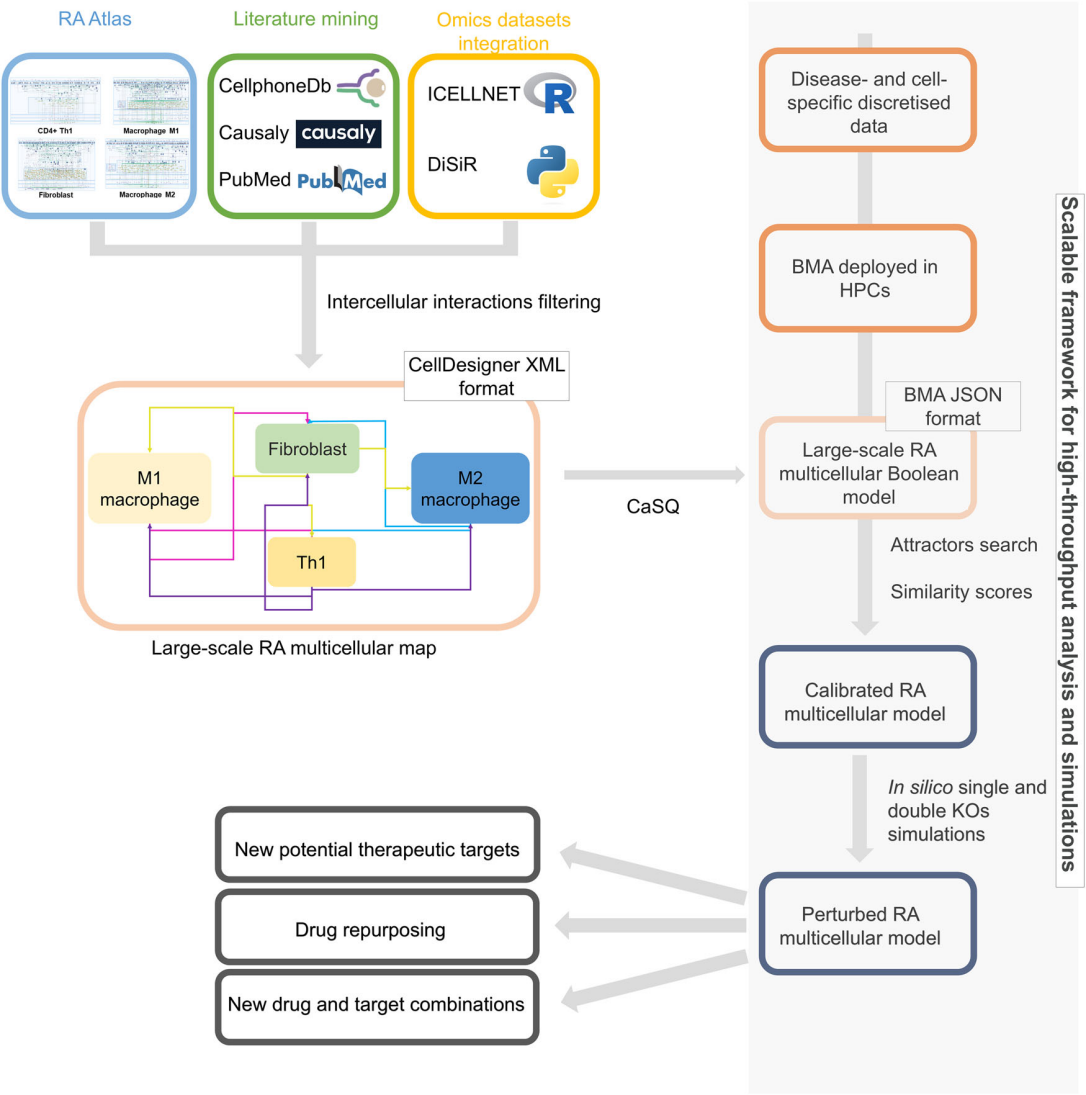
Workflow for the construction and calibration of the RA multi-cellular model. We used literature and database mining via several tools, like Causaly^68^ and CellphoneDB^69^, as well as keyword searches in PubMed, to retrieve the intercellular interactions already identified in the published literature. We also integrated omics datasets via ICELLNET^70^ and DiSiR^71^ to identify statistically significant interactions observed in these datasets. We filtered the retrieved interactions to cross-validate the results and only keep the most reliable ones. We used those filtered interactions to connect the RA cell-specific maps and generate the RA multi-cellular map. The resulting map in CellDesigner XML format is converted to an executable Boolean model using the CaSQ tool^21^. The BMA tool^22^ is then deployed on a high-performance computing cluster to identify all the model’s attractors. These attractors are filtered to keep only the steady states. Next, the filtered steady states are validated: Differentially expressed biomolecules in the model are identified using literature mining and transcriptomic data analysis. The identified biomolecule expressions are discretised and converted to a binary vector of experimentally observed Boolean values. After that, similarity scores are computed to describe the ability of the filtered steady states to reproduce the experimentally observed values. The steady states with the highest score are selected; their average vector represents the calibrated model’s state. The calibrated multi-cellular model is used to perform in silico simulations and identify potential new therapeutic targets and drug combinations, and to propose potential drug repurposing.

Figure 6 illustrates the workflow we developed to construct and calibrate the RA multi-cellular model.

### Identification of cell-cell interactions using literature and database mining

We used Causaly^68^ to uncover intercellular relationships in published literature. Causaly is a biomedical discovery research tool that uses advanced artificial intelligence, machine-reading literature across millions of academic publications, and distils the evidence into a knowledge graph.

We also used PubMed to retrieve cell-cell communication between the cell types of interest. Relevant keywords and key sentences like ‘M1 macrophage fibroblast interactions in rheumatoid arthritis’, ‘macrophage Th1 activation in RA’, and ‘M2 macrophage Th1 inhibition in RA’ among many others were used to filter the literature abstracts and studies in PubMed. Additionally, peer-reviewed articles concerning RA and their bibliography were searched, and information was mined.

We also used the CellPhoneDB database^69^, a publicly available repository of manually curated receptors, ligands, and their interactions, to identify the intercellular interactions occurring through ligands and receptors expressed by the four cell types represented in the RA-Atlas.

### Identification of cell-cell interactions using omics data

We used transcriptomic data to identify cell-cell interactions via the dedicated tools ICELLNET^70^ and DiSiR^71^. ICELLNET integrates an extensive database of manually curated ligand-receptor interactions collected from the literature and public databases. ICELLNET contains fewer interactions than existing databases but is very specific and exhaustive for cytokine interactions that play a critical role in RA development. DiSiR uses a user-defined putative list of ligand-receptor interactions. To run DiSiR, we used the ICELLNET database as a putative list of ligand-receptor interactions, some at the subunit level.

To cover both outward and inward communication between the four cell types of interest, we selected three single-cell RNA-seq datasets. Detailed descriptions of these datasets are available in the Datasets section. Table 6 summarises the pairs of gene expression datasets that we used to infer cell-cell communication between RA macrophages, RA fibroblast cell types and RA CD4 + Th1 cells.

**Table 6:** Pairs of datasets used to infer inward and outward cell-cell communication between RA macrophages, RA fibroblast and RA CD4 + Th1 cells

Interacting cell-types	Number of pairs of datasets	Cell source 1	Cell source 2
Macrophage ↔ Fibroblast	4	SDY998	SDY998
		E_MTAB_8322	SDY998
		SDY998	GSE109449
		E_MTAB_8322	GSE109449
Fibroblast ↔ CD4 + Th1	2	SDY998	SDY998
		GSE109449	SDY998
Macrophage ↔ CD4 + Th1	2	SDY998	SDY998
		E_MTAB_8322	SDY998

The first column lists the bidirectional interacting pairs of cell types represented in the RA multi-cellular model. Each pair is linked to a specific number of datasets, shown in the second column, used to identify its corresponding cell-cell communication. The third and fourth columns display the identifiers of the datasets used for the first and second cell types in each pair, respectively.

Synovial samples of such cell types are scarce and unavailable within the same dataset. The SDY998 dataset was the only one containing synovial fibroblasts, synovial T cells and synovial monocytes coming from RA patients. Therefore, we combined cells that come from different experimental designs. We used ComBat^72^ to reduce the resulting batch effect. Selected and processed datasets were then utilised to run both ICELLNET and DiSiR tools, first with “Cell source 1” and then with “Cell source 2” as sending cells. Only interactions with statistically significant *p*-values (FDR threshold equal to 0.05) identified using DiSiR and ICELLNET were kept.

We also filtered the interactions and only kept the ones identified with at least two different pairs of datasets or two different approaches (literature mining- and omics databases).

### Construction of the RA multi-cellular map in CellDesigner

The previously filtered cell-cell interactions were used to connect the RA cell-specific molecular interaction maps of the RA Atlas^16^. These maps are built in the Systems Biology Markup Language (SBML) format^73^ using CellDesigner^74^ and are compliant with the Systems Biology Graphical Notation (SBGN)^75^. They cover cell-specific signalling pathways, gene regulations, molecular processes and phenotypes involved in RA’s pathogenesis. Biomolecules and reactions in these maps are manually curated and extensively annotated through PubMed IDs, DOI, GEO and KEGG identifiers, following MIRIAM (Minimum Information Required In The Annotation of Models) standards^76^.

We used CellDesigner, a diagram editor tool for gene-regulatory and biochemical networks, which links interacting ligands and receptors. Ligands produced by the sending cell are transported via Transport arrows to the extracellular space of the receiving cell. The ligand will then bind to its corresponding receptor and form a ligand/receptor complex that can induce signal transduction in the cytoplasm of the receiving cell. When cell-cell interactions occur through cell-cell contact, Heterodimer Complex Association is used instead in CellDesigner to bind transmembrane proteins with their corresponding receptors.

### Generation and calibration of the RA cell-specific models

An efficient computational framework for analysing, calibrating, and validating large-scale Boolean models has recently been published^20^. In this framework, molecular interaction maps built in CellDesigner XML format are converted to executable Boolean models using the CaSQ tool^21^. A new BMA tool^22^ version is then deployed on a high-performance computing cluster to identify all the models’ attractors. Attractors depend on the external stimuli the model receives from its environment. These stimuli are modelled as inputs (i.e. nodes with no upstream regulation). They are not associated with any logical rule in the model; therefore, their values are user-defined. For the identification of all the attractors of the model, all the possible combinations of input values are generated, and the attractor search is performed for each input combination. These attractors are filtered to keep only the steady states. Differentially expressed biomolecules in the models are identified and converted to a binary vector of experimentally observed Boolean values to filter the resulting steady states further. After that, similarity scores are computed to describe the ability of the filtered steady states to reproduce the experimentally observed values. The steady states with the highest score are selected; their average vector represents the calibrated model’s state. This framework has already been applied to the RA M1 and M2 macrophage maps of the RA-Atlas. The associated results are described in the original publication and are used in our work^20^.

In this section, we apply N. Zerrouk et al.‘s methodology to the remaining cell-specific maps of the Atlas, namely the RA fibroblast and the RA Th1 maps. Phenotypes are particular nodes in these maps. Following N. Zerrouk et al.‘s work, we divided them into two categories. The first one corresponds to cell-specific phenotypes. They describe the cellular outcomes of each cell type of interest, like proliferation and apoptosis. The second category is not specific to a particular cell type. It corresponds to the RA joint’s cellular signals and biological conditions like inflammation, angiogenesis, and matrix degradation. We also updated the maps by looking for duplicates, removing them whenever they were found and correcting the signalling pathways accordingly.

We first focused on regulating the cell-specific phenotypes. As the second phenotype category describes the RA joint’s biological conditions and is influenced by several cell types, its regulation will be tested and validated when calibrating the RA multi-cellular model. We used the export option in CaSQ via the argument -u to identify all the nodes upstream of these phenotypes. The nodes not regulating the phenotypes of interest were not considered at this point.

Given the high number of inputs in the inferred Boolean models, we deployed BMA in a machine with 96 single-core CPUs and 768 GBs to compensate for the lack of computational power. We identified all the models’ attractors and only kept the steady states. We validated their behaviours using literature mining and gene expression datasets analysis. We extracted information from published literature regarding the differential expression of the models’ components. We curated the extracted information to keep it specific to both RA disease and the relevant cell type. We also integrated RA cell-specific gene expression datasets (*see Datasets section*). We discretised the differentially expressed molecules’ expressions: molecules that were overexpressed were linked to the value 1, whereas molecules that were under-expressed were linked to the value 0. Supplementary Tables 4 and 5 list these differentially expressed molecules in each cell-specific model and their observed Boolean values in the literature and/or gene expression datasets. The average vector over the steady states with the highest score defines the calibrated state of the models.

### Generation and calibration of the RA multi-cellular model

We used CaSQ to convert the RA multi-cellular interaction map to a Boolean model. To calibrate the resulting multi-cellular model, we first combined the cell-specific models’ calibrated states (including the calibrated states of the RA M1 and M2 macrophage models that we retrieved from^20^ via the addition of intercellular interactions present in the multi-cellular model. Then, we applied the previous framework to the remaining part of the multi-cellular model that is not upstream of the cell-specific phenotypes and that was not analysed during the cell-specific models’ calibration.

We used the BMA tool deployed to a machine with 96 single-core CPUs and 768 GB of RAM to run the attractors’ search and filter out the cycles. To calibrate the entirety of the multi-cellular model, we extracted information from published literature regarding the differential expression of these remaining biomolecules. We curated the extracted information to keep it specific to both RA disease and the relevant cell type. We also integrated additional DEGs from the same gene expression datasets we used for the cell-specific models’ calibration. Supplementary Table 8 describes the additional differentially expressed biomolecules identified in omics datasets and literature presenting this remaining part of the multi-cellular model. We calculated the similarity score between the list of differentially expressed molecules and their matching nodes in each steady state. Steady states with the highest similarity score were selected to calculate their mean vectors.

### Datasets

Regarding the RA macrophage cell type, we used the GSE97779 dataset^77^. It is a publicly available microarray dataset from the GEO database^78^. The dataset contains nine RA synovial macrophage samples from nine patients and five peripheral blood monocyte-derived macrophage samples from five healthy donors. In this work, we reused the normalised gene expressions and the list of differentially expressed genes (DEGs) between RA and healthy control samples provided in the RA-Atlas publication^16^. We also used E_MTAB_8322^32^, an RNA-seq single-cell dataset publicly available in the ArrayExpress database^79^. It contains synovial samples coming from five treatment-naïve RA, six treatment-resistant RA, six in sustained remission and four patients with UPA (Early undifferentiated arthritis). Four healthy donor synovial tissues were included as control. The macrophage population was identified using FACS sorting. Quality control of the dataset involved removing cells with less than 500 expressed genes and removing weakly expressed genes. In this work, we normalised gene expressions using Seurat’s *NormalizeData* function. We only used the gene expression matrix of the five treatment naïve RA patients (5815 macrophage cells).

Regarding the RA fibroblast cell type, we used the GSE109449 dataset^80^. It is a single-cell (sc) RNA-seq dataset in the GEO database. It contains 384 freshly isolated synovial fibroblasts in two RA and two osteoarthritis (OA) patients. In this work, we reused the normalised gene expressions and the list of DEGs between RA and healthy control samples provided in the RA-Atlas publication. SDY998^81^ is a single-cell RNA-seq dataset from the Immport database^82^ containing 19 samples from RA patients and two synovial samples from OA patients, including four cell types: 1142 B cells, 1844 fibroblasts, 750 monocytes, and 1529 T cells. In this work, we used the gene expression of the CD4 + Th1 cluster and the list of DEGs between the RA Th1 cluster and RA naive CD4 + T cells provided in the RA-Atlas publication.

## Supplementary information

Supplementary material

## Data Availability

The datasets GSE97779 and GSE109449, available through the NCBI GEO database, were utilized in this work. Additionally, the E_MTAB_8322 dataset from ArrayExpress database and the SDY998 dataset from ImmPort database were also used. The RA multi-cellular model is available in the BioModels repository under the identifier MODEL2408030001. For an optimal visual exploration of the maps, all cell-specific maps are available as online interactive maps on the standalone web server MINERVA at https://ramap.uni.lu/minerva/.

## Code availability

Scripts and files generated are available at https://github.com/Sanofi-Public/Large_Scale_Multicellular_Modelling_For_RA.
