# Supplementary material for: Building a modular and multi-cellular virtual twin of the synovial joint in Rheumatoid Arthritis
Source: NPJ Digit Med. 2024 Dec 24;7:379. doi: 10.1038/s41746-024-01396-y (PMC11668869; doi:10.1038/s41746-024-01396-y)
Supplement: Supplementary file 1 — Supplementary material [file 41746_2024_1396_MOESM1_ESM.pdf]

Building a modular and multi-cellular virtual twin of the synovial joint in Rheumatoid Arthritis

Supplementary Data

Supplementary Table 1. Cell-cell interactions between RA macrophage and RA fibroblast in the synovium.

Ligand/ receptor complexes	Sending cell	Receiving cell	Source		
			Literature mining (PMID)	CellPhoneDB database	Omics datasets pairs
CXCL1/ CXCR1	Fibroblast	M1 macrophage	33087182	Absent	(SDY998, SDY998)<br>(GSE109449, SDY998)
CCL2/ CCR2	Fibroblast	M1 macrophage		Present	(SDY998, SDY998)
CSF2/ CSF2RA+ CSF2RB	Fibroblast	M1 macrophage	1700731<br>27813830<br>29997624	Present	
TNFSF11/ TNFRSF11 A	Fibroblast	M1 macrophage		Absent	(GSE109449, SDY998)<br>(SDY998, SDY998)<br>(GSE109449, E_MTAB_8322)
TNFA/ TNFRSF1A	Fibroblast	M1 macrophage	29997624<br>28807007<br>27813830<br>25057003	Present	
IL1B/IL1R	Fibroblast	M1 macrophage	28807007<br>26883280<br>29997624	Present	(GSE109449, SDY998)<br>(SDY998, E_MTAB_8322)
JAG1/ NOTCH1	Fibroblast	M1 macrophage		Present	(SDY998, SDY998)<br>(GSE109449, E_MTAB_8322)
GAL/GALR 2	Fibroblast	M1 macrophage		Absent	(SDY998, SDY998)<br>(GSE109449, SDY998)
COL4A4/ ITGA1+ ITGB1	Fibroblast	M1 macrophage		Present	(GSE109449, SDY998)<br>(SDY998, SDY998)
HLA-B/ LILRB1	Fibroblast	M1 macrophage		Absent	(SDY998, SDY998)<br>(SDY998, E_MTAB_8322)<br>(GSE109449, E_MTAB_8322)
IFNE/ IFNAR1+ IFNAR2	Fibroblast	M1 macrophage		Present	(SDY998, E_MTAB_8322)
IL6/IL6R+ IL6ST	Fibroblast	M1 macrophage	29997624<br>35663975	Present	(SDY998, SDY998)
IL12/ IL12RB1+ IL12RB2	Fibroblast	M1 macrophage		Present	(GSE109449, SDY998)

HLA-B/ LILRB1	Fibroblast	M2 macrophage		Absent	(SDY998, SDY998) (SDY998, E_MTAB_8322) (GSE109449, E_MTAB_8322)
VEGFC/ VEGFR3	Fibroblast	M2 macrophage		Present	(GSE109449, SDY998) (SDY998, SDY998)
GAL/GALR2	Fibroblast	M2 macrophage		Absent	(SDY998, SDY998) (GSE109449, SDY998)
SEMA4D/ PLXNB2+ MET	Fibroblast	M2 macrophage		Present	(GSE109449, E_MTAB_8322)
EFNB1/ EPHB1	Fibroblast	M2 macrophage		Present	(SDY998, SDY998)
WNT5B/ FZD1+LRP5	Fibroblast	M2 macrophage	30022048	Present	(GSE109449, SDY998)
CSF1/CSF1R	Fibroblast	M2 macrophage	27383913	Present	(SDY998, E_MTAB_8322)
IL34/ CSFR1R	Fibroblast	M2 macrophage		Present	(GSE109449, E_MTAB_8322) (GSE109449, SDY998)
TGFB/ TGFBR1+ TGFBR2	Fibroblast	M2 macrophage	29997624 31068444	Present	(GSE109449, SDY998)
PRL/PRLR	Fibroblast	M2 macrophage		Absent	(SDY998, SDY998) (GSE109449, SDY998)
COL4A4/ ITGB1+ ITGA1	Fibroblast	M2 macrophage		Present	(GSE109449, SDY998) (SDY998, SDY998)
SEMA3A/ PLXNA1	Fibroblast	M2 macrophage		Present	(GSE109449, SDY998) (SDY998, E_MTAB_8322)
GAS6/ MERTK	Fibroblast	M2 macrophage		Absent	(SDY998, SDY998) (GSE109449, SDY998)
CD40L/ CD40	M1 macrophage	Fibroblast		Absent	(SDY998, GSE109449) (SDY998, SDY998)
JAG1/ NOTCH3	M1 macrophage	Fibroblast		Present	(SDY998, SDY998)
EDA/EDA2R	M1 macrophage	Fibroblast		Absent	(SDY998, GSE109449) (E_MTAB_8322, SDY998)
ICAM1/ ITGB2+ ITGAL	M1 macrophage	Fibroblast		Present	(SDY998, SDY998) (E_MTAB_8322, SDY998)
IL18/IL18R	M1 macrophage	Fibroblast		Present	(E_MTAB_8322, GSE109449) (E_MTAB_8322, SDY998) (SDY998, GSE109449)
IL1B/ IL1R1	M1 macrophage	Fibroblast	27383913 31178859 31068444	Present	(E_MTAB_8322, GSE109449)
TNFA/ TNFRSF1B	M1 macrophage	Fibroblast	27383913 31178859	Present	
CXCL10/ CXCR3	M1 macrophage	Fibroblast		Absent	(SDY998, SDY998) (E_MTAB_8322, SDY998)

					(E_MTAB_8322, GSE109449)
IFNG/ IFNGR1+ INFGR2	M1 macrophage	Fibroblast		Present	(SDY998, SDY998) (SDY998, GSE109449) (E_MTAB_8322, SDY998)
FASL/FAS	M1 macrophage	Fibroblast		Absent	(SDY998, SDY998) (SDY998, GSE109449)
CCL5/CCR 5	M1 macrophage	Fibroblast		Present	(SDY998, GSE109449)
HBEGF/ EGFR	M1 macrophage	Fibroblast	31068444	Present	
AREG/EGF R	M1 macrophage	Fibroblast		Present	(SDY998, SDY998) (E_MTAB_8322, GSE109449)
SEMA4A/ PLXNB1+ MET	M1 macrophage	Fibroblast	26303122	Present	(E_MTAB_8322, SDY998)
FASL/FAS	M2 macrophage	Fibroblast		Absent	(SDY998, SDY998) (SDY998, GSE109449)
CCL18/ PITPNM3	M2 macrophage	Fibroblast		Absent	(SDY998, GSE109449) (SDY998, SDY998) (E_MTAB_8322, GSE109449)
EDA/ EDA2R	M2 macrophage	Fibroblast		Absent	(SDY998, GSE109449) (E_MTAB_8322, SDY998)
JAG1/ NOTCH3	M2 macrophage	Fibroblast		Present	(SDY998, SDY998)
IL10/ L10RA+ IL10RB	M2 macrophage	Fibroblast		Present	(SDY998, GSE109449) (SDY998, SDY998)
PDGFC/ PDGFRB	M2 macrophage	Fibroblast		Present	(SDY998, SDY998) (E_MTAB_8322, GSE109449)
HBEGF/ EGFR	M2 macrophage	Fibroblast	31068444	Present	
COL4A3/ ITGA4+ ITGB1	M2 macrophage	Fibroblast		Present	(SDY998, SDY998) (E_MTAB_8322, SDY998) (SDY998, GSE109449)
SEMA7A/ ITGA4+ ITGB1	M2 macrophage	Fibroblast		Absent	(SDY998, GSE109449) (SDY998, SDY998)
VEGFA/ VEGFR	M2 macrophage	Fibroblast		Absent	(SDY998, SDY998) (E_MTAB_8322, SDY998)
TGFB1/ TGFBR1	M2 macrophage	Fibroblast		Present	(SDY998, GSE109449) (E_MTAB_8322, GSE109449)
SEMA4A/ PLXNB1+ MET	M2 macrophage	Fibroblast	26303122	Present	(E_MTAB_8322, SDY998)
CD40L/ CD40	M2 macrophage	Fibroblast		Absent	(SDY998, GSE109449) (SDY998, SDY998)

Supplementary Table 2. Cell-cell interactions taking place between RA macrophage and RA CD4+ Th1 in the synovium.

Ligand/receptor complexes	Sending cell	Receiving cell	Source		
			Literature mining	CellPhoneDB database	Omics dataset(s)
IFNG/ IFNGR1+ IFNGR2	Th1	M1 macrophage	25329467	Present	(SDY998, E_MTAB_8322)
CXCL13/ACKR4	Th1	M1 macrophage		Absent	(SDY998, SDY998) (SDY998, E_MTAB_8322)
FASL/FAS	Th1	M1 macrophage		Absent	(SDY998, SDY998) (SDY998, E_MTAB_8322)
COL4A5/ ITGA1+ITGB1	Th1	M1 macrophage		Present	(SDY998, SDY998) (SDY998, E_MTAB_8322)
TNFSF11/ TNFRSF11A	Th1	M1 macrophage	16220542	Absent	(SDY998, SDY998)
CD40L/ ITGB1+ITGA1	Th1	M1 macrophage		Absent	(SDY998, SDY998) (SDY998, E_MTAB_8322)
SEMA4A/ PLXNB2+MET	Th1	M2 macrophage		Present	(SDY998, E_MTAB_8322)
CXCL13/ACKR4	Th1	M2 macrophage		Absent	(SDY998, SDY998) (SDY998, E_MTAB_8322)
FASL/FAS	Th1	M2 macrophage		Absent	(SDY998, SDY998) (SDY998, E_MTAB_8322)
CD40L/ITGB1+ ITGA1	Th1	M2 macrophage		Absent	(SDY998, SDY998) (SDY998, E_MTAB_8322)
COL4A5/ ITGB1+ITGA1	Th1	M2 macrophage		Present	(SDY998, E_MTAB_8322)
TGFB1/TGFBR1+ TGFBR2	Th1	M2 macrophage		Present	(SDY998, SDY998)
IL12/IL12Rb1+ IL12Rb2	M1 macrophage	Th1	26635790	Present	
IFNG/IFNGR1+ IFNGR2	M1 macrophage	Th1		Present	(SDY998, SDY998) (E_MTAB_8322, SDY998)
CCL4/CCR5	M1 macrophage	Th1		Present	(E_MTAB_8322, SDY998)
CXCL10/CXCR3	M1 macrophage	Th1		Absent	(SDY998, SDY998) (E_MTAB_8322, SDY998)

IL18/IL18R1+ IL18RAP	M1 macrophage	Th1	10562301	Present	
LGALS9/TIM3	M1 macrophage	Th1		Absent	(SDY998, SDY998) (E_MTAB_8322, SDY998)
ICOSLG/ICOS	M1 macrophage	Th1		Present	(E_MTAB_8322, SDY998)
HLA-DP-DQ-DR/ TCR+CD3	M1 macrophage	Th1	30915067	Absent	(SDY998, SDY998)
HLA-DP-DQ-DR /LAG3	M1 macrophage	Th1		Absent	(SDY998, SDY998) (E_MTAB_8322, SDY998)
CXCL16/CXCR6	M1 macrophage	Th1	26635790	Absent	(E_MTAB_8322, SDY998)
CD40L/ITGAM+ ITGB2	M1 macrophage	Th1		Absent	(SDY998, SDY998) (E_MTAB_8322, SDY998)
SEMA4A/PLXNB 1	M1 macrophage	Th1		Present	(SDY998, SDY998)
JAG1/NOTCH1	M1 macrophage	Th1		Present	(SDY998, SDY998)
CD28/CD86	M1 macrophage	Th1	29868020	Present	
LTA/TNFRSF14	M1 macrophage	Th1		Absent	(SDY998, SDY998) (E_MTAB_8322, SDY998)
CCL2/CCR2	M1 macrophage	Th1	26635790	Present	
COL4A3/ ITGA4+ITGB7	M2 macrophage	Th1		Present	(SDY998, SDY998) (E_MTAB_8322, SDY998)
ICOSLG/ICOS	M2 macrophage	Th1		Present	(E_MTAB_8322, SDY998)
HLA-DP-DQ-DR /TCR+CD3	M2 macrophage	Th1	30915067	Absent	(SDY998, SDY998)
HLA-DP-DQ-DR /LAG3	M2 macrophage	Th1		Absent	(SDY998, SDY998) (E_MTAB_8322, SDY998)
CXCL16/CXCR6	M2 macrophage	Th1	26635790	Absent	(E_MTAB_8322, SDY998)
CD40L/ITGAM+ ITGB2	M2 macrophage	Th1		Absent	(SDY998, SDY998) (E_MTAB_8322, SDY998)
SEMA4A/PLXNB 1	M2 macrophage	Th1		Present	(SDY998, SDY998)
JAG1/NOTCH1	M2 macrophage	Th1		Present	(SDY998, SDY998)
CD28/CD86	M2 macrophage	Th1	29868020	Present	

MIF/CD74	M2 macrophage	Th1		Absent	(SDY998, SDY998) (E_MTAB_8322, SDY998)

Supplementary Table 3. Cell-cell interactions taking place between RA CD4+ Th1 and RA fibroblast in the synovium.

Ligand/receptor complexes	Sending cell	Receiving cell	Source		
			Literature mining	CellPhoneDB database	Omics dataset(s)
CCL5/CCR5	Th1	Fibroblast		Present	(SDY998, SDY998)
COL4A5/ ITGA5+ITGB1	Th1	Fibroblast		Absent	(SDY998, SDY998) (SDY998, GSE109449)
TGFB1/TGFBR1	Th1	Fibroblast		Present	(SDY998, SDY998)
SEMA4A/ PLXNB1+MET	Th1	Fibroblast		Present	(SDY998, GSE109449)
CD40L/CD40	Th1	Fibroblast	35844494 15077296	Absent	(SDY998, SDY998) (SDY998, GSE109449)
ICAM1/ ITGB2+ITGAL	Th1	Fibroblast	27623446	Present	
TNFSF11/ TNFRSF11A	Th1	Fibroblast		Absent	(SDY998, SDY998) (SDY998, GSE109449)
IL18/IL18R	Th1	Fibroblast		Present	(SDY998, GSE109449)
CXCL13/CXCR3	Th1	Fibroblast		Absent	(SDY998, SDY998) (SDY998, GSE109449)
MIF/CXCR4	Th1	Fibroblast		Absent	(SDY998, SDY998) (SDY998, GSE109449)
IFNG/ IFNGR1+INFGR2	Th1	Fibroblast	32047926	Present	(SDY998, SDY998)
FASL/FAS	Th1	Fibroblast		Absent	(SDY998, SDY998) (SDY998, GSE109449)
CCL5/CCR5	Fibroblast	Th1		Present	(GSE109449, SDY998)
IL7R/IL2RG	Fibroblast	Th1	35844494	Absent	(SDY998, SDY998) (GSE109449, SDY998)
TNFA/TNFRSF1B	Fibroblast	Th1		Present	(SDY998, SDY998)
GAS6/MERTK	Fibroblast	Th1		Absent	(SDY998, SDY998) (GSE109449, SDY998)
CD84/CD84	Fibroblast	Th1		Absent	(SDY998, SDY998) (GSE109449, SDY998)
CCL2/CCR2	Fibroblast	Th1		Present	(SDY998, SDY998)
MIF/CD74	Fibroblast	Th1		Absent	(SDY998, SDY998) (GSE109449, SDY998)
LTA/TNFRSF14	Fibroblast	Th1		Absent	(SDY998, SDY998)

					(GSE109449, SDY998)
JAG1/NOTCH1	Fibroblast	Th1		Present	(GSE109449, SDY998) (SDY998, SDY998)
SEMA4D/PLXNB 1	Fibroblast	Th1		Present	(SDY998, SDY998) (GSE109449, SDY998)
VCAM1/ ITGA4+ITGB7	Fibroblast	Th1	27623446 35844494	Present	
ICOSLG/ICOS	Fibroblast	Th1		Present	(SDY998, SDY998)
CXCL10/CXCR3	Fibroblast	Th1		Absent	(SDY998, SDY998) (GSE109449, SDY998)
IL10/ IL10RA+IL10RB	Fibroblast	Th1		Present	(SDY998, SDY998)
LGALS9/TIM3	Fibroblast	Th1		Absent	(SDY998, SDY998) (GSE109449, SDY998)

Supplementary Table 4. The list of differentially expressed genes present in the RA fibroblast model that we identified using literature search and omics data analysis. The first column contains the DEGs HGNC names. The second and fifth columns contain their corresponding Boolean values (after data discretization) observed in GSE109449 dataset and literature respectively.

DEG	Boolean value in GSE109449	Adjusted p_value	logFC	Boolean value in literature	Reference
ADCY8	0	0.002	-3,02		
AREG				1	18439312
ARHGEF 2				1	17515956
BAX				0	23421940
BCL2				1	17515956
BCL2L1				1	28118944
BCL2L11				0	2811894 4
BID	0	0.004	-1,47		
CASP3	0	4.524e-05	-0,72		
CCL18				1	1174539 6
CCL2	1	0.001	2,19	1	33330982
CCL5				1	9756723
CCR2				1	11673556
CCR5				1	11673556
CD14				1	18452992
CD40				1	10799861
CFLAR				1	15593196
Cgas				1	26819496
COMP	1	5.036e-18	4,33	1	19652761

CSF2				1	31142839
CSF2RA				1	24936585
CSF2RB				1	24936585
CSK				1	17515956
CXCL10				1	28148302
CXCL13				1	27102921
CXCL8				1	11178128
CXCR2				1	20036936
CXCR3				1	21811993
CXCR4				1	32782501
CYCS	0	0.004	-0,687		
FN1				1	36225320
EDA				1	7748223
EGF				1	18439312
EGFR				1	18439312
GAB2	0	0.0004	-1,35		
FAS				1	11169523
FASLG	1	0.022	0,14	1	11169523
FGF1	1	0.004	1,76	1	16893535
FGFR4	1	0.039	0,84	1	8651984
IGF1R	0	0.003	-1,17		
FOS				1	7747113
FOXO1				0	24812285
FZD5				1	11315916
NRP1	0	0.001	-0,89		
HBEGF				1	31068444
ICAM1				1	17568789
IFNB				1	15878901
IFNG				1	31061532
IGF1				0	11934980
RHOA	0	0.004	-0,59		
IL10				1	20001767
IL10RA				1	27626941
IL10RB				1	27626941
IL17				1	23858337
IL18				1	10562301
IL18R				1	17530707
IL1A				1	3027299 6
IL1B				1	3027299 6
DUSP7	1	0.003	0,85		
IL6				1	32718086
IRAK4				1	18452992
IRF5				1	26315890
EPHB2	1	0.013	1,75		
GAB1	1	0.0003	1,55		

IL1R1	1	0.01	1,16		
JUN				1	7747113
LY96				1	26352601
MAP2K3				1	14695331
MAP2K4				1	13130464
MAP2K6				1	14695331
MAP2K7				1	13130464
MAP3K1				1	22736089
ITGA4	1	0.0002	2,12		
MCL1				1	16339575
MDM2				1	26655743
MIF				1	12011381
MIR146A				0	29844864
MIR192				0	28321538
MIR650				0	28129626
MMP1				1	12379519
MMP13				1	11040455
MMP3				1	12379519
NFAT5				1	21717420
NFKB1				1	12010604
ITGA5	1	0.019	1,35		
P53				1	11169523
PDGFC	1	0.042	0,18	1	26976956
PDGFRB	1	3.556e-08	1,86	1	26976956
PITPNM3				1	23728190
MAP4K4	1	1.941e-06	1,86		
PTPN11				1	23335101
PLXNB1	1	0.0003	1,45		
PTPRC	1	0.012	1,36		
RANKL				1	10693864
RAC1	1	0.035	0,61		
RIPK1				1	32116107
SEMA4A				1	26303122
SEMA7A				1	28109308
SOCS3				1	30854695
SRC	1	0.003	1,78		
SST				0	9844773
SSTR				0	9844773
STAT1	1	0.006	1,79	1	25630235
STAT3				1	30477351
TAB2				1	18452992
TBK1				1	18452992
TCF19	1	0.030	1,76		
TGFB1				1	11966774
TGFBR1				1	17594488
TIRAP				1	18452992

TLR2				1	32256787
TLR4				1	32256787
TLR5				1	22661088
TNF				1	34588517
TNFR				1	16951485
TRADD				1	16951485
TRAF2				1	16951485
TRAF6				1	18452992
TXK	1	0.010	0,79		
VAV3	1	0.038	1,41		
VEGFA	1	0.002	1,55	1	18439312
WNT5A				1	11315916
YY1				1	26821827

Supplementary Table 5. The list of differentially expressed genes present in the RA CD4+ Th1 model that we identified using literature search and omics data analysis. The first column contains the DEGs HGNC names. The second and fifth columns contain their corresponding Boolean values (after data discretization) observed in SDY998 dataset and literature respectively.

DEG	Boolean value in SDY998	Adjusted p_value	logFC	Boolean value in literature	Reference
F2R	1	3,47E-13	3,77		
CCL2				1	1522232
CCR5	1	1,19E-16	3,81	1	10323208
CCL5	1	2,48E-11	6,45		
CCL4	1	1,12E-13	5,97		
CXCL10				1	28148302
CXCR3				1	27190305
CXCL16				1	16200580
EOMES	1	1,59E-44	5,67		
FGR	1	1,59E-18	6,4		
LAG3	1	1,46E-11	4,8		
HLA-DRB5	1	0,0003	5,52		
HLA-DQA1	1	4,19E-14	5,27		
HLA-DQB2	1	1,53E-08	4,94		
HLA-DQA2	1	4,44E-16	5,2		
HLA-DRB1	1	5,97E-09	3,97		
HLA-DPB1	1	6,87E-08	3,59		
IFNG	1	2,98E-15	5,06	1	8961900
IFNGR1				1	25708927
IFNGR2				1	25708927
IL12				1	32264938
IL12RB1				1	32264938
IL12RB2				1	32264938
IL18R1				1	14994387

IL18				1	14532149
IL9R				1	19723899
IL2R				1	32264938
IL9				1	26078482
TIM3	1	1,04E-19	7,35		
CD74	1	0,014	2,89		
IL27				1	17015723
IL27R				1	32518420
STAT1				1	33779079
STAT4				1	17804842
TBX21				1	3333523
TNFSF4	1	1,54E-09	5,96	1	11069062
VCAM1	1	2,97E-15	7,01		

Supplementary Table 6. List of nodes upstream the phenotypes of interest in the RA fibroblast model associated with their mean values over the fixpoints having the highest similarity score.

Nodes	Mean values
ADCY8	0
AREG	1
ARHGEF2	1
BAX_Fibroblast___Mitochondrion	0
BCL2	1
BCL2L1_rna	1
BCL2L11_Fibroblast___Cytoplasm	0
BID	0
CASP3	0
CCL18	1
CCL2_Fibroblast___secreted_components	1
CCL5_Fibroblast___secreted_components	1
CCR2_CCL2_complex	1
CCL5_CCR5_complex	1
LBP_CD14_complex	1
CFLAR_Fibroblast___Cytoplasm	1
cGAS	1
COMP	1
CRKL_phosphorylated	1
CSF2_Fibroblast___Extracellular_Space	1
CSF2RA_CSF2RB_complex	1
CSK	1
CXCL10_Fibroblast___Extracellular_Space	1
CXCL13	1
CXCL8_Fibroblast___Extracellular_Space	1
CXCR2_CXCL8_complex	1
CXCL10_CXCR3_complex	1

MIF_CXCR4_complex	1
CYCS	0
FN1_Fibroblast___secreted_components	0
EDA	1
EGF	1
EGFR	1
GAB2_phosphorylated	1
FAS_FASL_complex	1
FGF1	1
IGF1_IGF1R_complex	0
FOS	1
FOXO1	0
WNT_FRIZZLED_complex	1
FN1_ITGAV_complex	0
VEGFR_NRP1_complex	0
HBEGF	1
IFNb1	1
IFNG_IFNGR1_R2_complex	1
IGF1	0
RHOA	0
IL10RA_IL10RB_IL10_complex	1
IL17A_Fibroblast___secreted_components	1
IL18_Fibroblast___secreted_components	1
IL18_IL18R_complex	1
IL1A	1
IL1B_Fibroblast___secreted_components	1
DUSP7	1
IL6_Fibroblast___secreted_components	1
IRAK4_Fibroblast___Cytoplasm	1
IRF5_Fibroblast___Cytoplasm	1
EPHB2	1
GAB1	1
IL1R1	1
JUN_phosphorylated	1
LY96	1
MAP2K3_phosphorylated	1
MAP2K4_phosphorylated	1
MAP2K6_phosphorylated	1
MAP2K7_phosphorylated	1
MAP3K1_phosphorylated	1
ITGA4_ITGB1_complex	1
MCL1	1
MDM2	1
MIF	1
MIR146A_rna	0
MIR192_rna	0

MIR650_rna	0
MMP1	1
MMP13	1
MMP3	1
NFAT5_phosphorylated	1
NFKB_complex	1
ITGA5_ITGB1_col4a5_complex	1
TP53_phosphorylated	1
PDGFC	1
PDGFC_PDGFRB_complex	1
PITPNM3_CCL18_complex	1
MAP4K4_phosphorylated	1
PTPN11_phosphorylated	1
PLXNB1_MET_sema4a_complex	1
NECTIN3_PTPRC_NECTIN1_complex	1
RANK_RANKL_complex	1
RAC1_2	1
RIPK1	1
ITGA4_ITGB1_sema7a_complex	1
SOCS3	1
SRC_phosphorylated	1
sst	0
sst_sstr_complex	0
STAT1_Fibroblast___Cytoplasm	1
STAT3_Fibroblast___Cytoplasm	1
TAB1_TAB2_complex	1
TBK1_IKBKE_complex	1
TCF_LEF	1
TGFb1	1
TGFB1_TGFBR1_complex	1
TIRAP	1
LY96_TLR2_4_complex	1
TLR5	1
TNF_Fibroblast___secreted_components	1
TNF_TNFRSF1A_B_complex	1
TRADD	1
TRAF2	1
TRAF6	1
TXK	1
VAV1_2_3_phosphorylated	1
ADAMTS9_rna	0,5
AKT2	1
AKT2_phosphorylated	1
AMAP1	1
APAF1	0,5
apoptosis_fibroblast_phenotype	0

Apoptosome_complex	0
AREG_EGFR_complex	1
BAD	0
BAX_Fibroblast___Mitochondrion_active	0
BCL2L11_Fibroblast___Cytoplasm_active	0
BRAF_phosphorylated	0
CALCINEURIN	1
CASP8	0
CASP9	0,5
CAV1_rna	1
CCL2_Fibroblast___Extracellular_Space	1
CCL5_Fibroblast___Extracellular_Space	1
CD40LG_CD40_complex	1
Cell_chemotaxis_migration_fibroblast_phenotype	1
CFLAR_Fibroblast___Cytoplasm_active	1
col4a3	0,5
CREB1_phosphorylated	1
CSF2_Fibroblast___secreted_components	1
CSF2RA_CSF2RB_CSF2_complex	1
CTNNB_CK1A_AXIN_GSK3B_APC_complex	0
CTNNB1	0
CXCL10_Fibroblast___secreted_components	1
CXCL13_CXCR3_complex	1
CXCL8_Fibroblast___secreted_components	1
CXCR3	1
DAXX	1
DOCK2	0,5
DOCK2_CRKL_complex	0,5
DVL1_phosphorylated	1
EDA_EDA2R_complex	1
EGF_EGFR_complex	1
FADD	1
FASLG	1
FGF1_FGFR4_complex	1
FN1_Fibroblast___Extracellular_Space	0
FOXO3	0
GNAI3	1
GRB2	1
HBEGF_EGFR_complex	1
HOMODIMER_space_STAT1	1
ICAM1_ITGB2_ITGAL_complex	1
IFNa1	1
IFNa1_B1	1
IFNA1_B1_IFNAR1_R2_complex	1
IFNB1_rna	1
IKBA_NFKB1_RELA_complex	1

IKBKB_phosphorylated	1
IKK_complex	1
IKK1_IKK2_complex	0,25
IKK1_phosphorylated	0,5
IKK2_phosphorylated	0,5
IL17A_Fibroblast___Extracellular_Space	1
IL17A_IL17RA_complex	1
IL18_Fibroblast___Extracellular_Space	1
IL1A_IL1R1_complex	1
IL1B_Fibroblast___Extracellular_Space	1
IL1B_IL1R1_complex	1
IL6_Fibroblast___Extracellular_Space	1
IL6_IL6R_complex	1
IL6_IL6ST_complex	1
IRAK1_Fibroblast___Cytoplasm	1
IRAK1_Fibroblast___Cytoplasm_active	1
IRAK1_IRAK4_complex	1
IRAK4_Fibroblast___Cytoplasm_active	1
IRF3_phosphorylated	1
IRF5_Fibroblast___nucleus	1
IRF9	1
ISGF3_complex	1
ITGA4_ITGB1_col4a3_complex	0,5
JAK1	0
JAK2	0
JAK3	0
LCK_phosphorylated	0,5
LTBP1	0
MAP2K1_phosphorylated	1
MAP3K14_phosphorylated	1
MAP3K2_3_4	0,5
MAP3K5_phosphorylated	1
MAP3K7_phosphorylated	1
MAP3K8_phosphorylated	1
MAPK1_empty	0
MAPK1_phosphorylated	0
MAPK14_phosphorylated	1
MAPK3_complex	0,5
MAPK3_phosphorylated	1
MAPK8_phosphorylated	0,5
MAPK9_phosphorylated	1
MAPKAPK2_phosphorylated	1
MDM2_phosphorylated	1
MIR10a_rna	0
MIR338_5P_rna	0,5
MIR451A_rna	0

MYD88	1
NFKB_N_complex	1
NFKB1_MAP3K8_complex	1
ngef	1
NRAS	0
p38MAPK_empty	1
p38MAPK_phosphorylated	0
PI3_4_5_P__3_simple_molecule	0,5
PI4_5_P__2_simple_molecule	1
PIK3R5_phosphorylated	1
PP2A	0
PRKACA	0
PRKCQ	0,5
PRKG1	1
proliferation_survival_fibroblast_phenotype	1
PTEN	0,5
PTK2B_phosphorylated	1
PTPN6	0,5
pyk2_phosphorylated	1
RAB5A	1
RAF1	0,5
rasa1	1
RELA_NFKB1_NFKBIE_complex	1
RIPK1_TRAF6_complex	1
RPS6KB1_phosphorylated	1
SARM1	0
sema7a	1
SH2D1A	0,5
SHC2_phosphorylated	1
SHP2_GRB2_complex	1
SOS1	1
STAT1_Fibroblast___nucleus	1
STAT1_STAT2_complex	1
STAT2	1
STAT3_Fibroblast___nucleus	1
TAB1_TAB2_TAK1_complex	1
TAB1_TAB2_TRAF6_complex	1
tBID	0
TGFB1_Fibroblast___Extracellular_Space	1
TGFB1_Fibroblast___Extracellular_Space_active	1
TICAM1	1
TICAM1_TICAM2_complex	1
TICAM2	1
TIRAP_MYD88_complex	1
TNF_Fibroblast___Extracellular_Space	1
TNFRSF10A_rna	1

TNFRSF10B_rna	1
TNFSF11_Fibroblast___Extracellular_Space	1
TNFSF11_Fibroblast___secreted_components	1
TP73_phosphorylated	0
TRAF2_TRAF5_complex	1
TRAF3	1
TRAF3_TRAF6_complex	1
TRAF3IP2_phosphorylated	1
TRAF6_phosphorylated	1
TYK2	1
VEGFa_Fibroblast___Extracellular_Space	1
VEGFa_Fibroblast___secreted_components	1
VEGFA_rna	1
VEGFA_VEGFR_NRP1_complex	0
WNT5A	1
YWHAQ	0
YY1	1
ZC3H12A	0

Supplementary Table 7. List of nodes upstream the phenotypes of interest in the RA CD4+ Th1 model associated with their mean values over the fixpoints having the highest similarity score.

Nodes	Mean values
AGT	1
AGT_F2R_complex	1
AKT1	1
apoptosis_TH1_phenotype	0
ARHGEF2	1
BCL2L11	0
CCL2	1
CCL2_CCR2_complex	1
CCL4_5_CCR5_complex	1
CCL4_TH1___extracellular_space	1
CCL4_TH1___secreted_components	1
CCL5_TH1___extracellular_space	1
CCL5_TH1___secreted_components	1
CCR5	1
CD28_CD86_complex	0,5
CD32b_igG_complex	0
CD84_CD84_complex	0,5
Cell_chemotaxis_migration_TH1_phenotype	1
COL4A3_ITGA4_ITGB7_complex	0,5
CREB1_phosphorylated	1
CXCL10_CXCR3_complex	1
CXCL10_TH1___extracellular_space	1

CXCL10_TH1___secreted_components	1
CXCL16	1
CXCL16_CXCR6_complex	1
EOMES	1
FGR	1
GAS6	0,5
gas6_mertk_complex	0,5
GNA12_GNA13_complex	1
GNAI_GNB_GNG_complex	1
GNAI_TH1___cytoplasm	1
GNAI_TH1___cytoplasm_active	1
GNB_GNG_complex	1
GRB2	1
HLA_DP_DQ_DR_LAG3_complex	1
HLA_DP_DQ_DR_TCR_CD3_complex	1
icoslg_icos_complex	0,5
IFNG_IFNGR1_IFNGR2_complex	1
IFNg_TH1___extracellular_space	1
IFNg_TH1___secreted_components	1
IFNGR1_IFNGR2_complex	1
igG	0
IKK_complex	1
IL10_IL10RA_IL10RB_complex	1
IL12_TH1___extracellular_space	1
IL12_TH1___secreted_components	1
IL12Rb1_IL12Rb2_complex	1
IL12Rb1_IL12Rb2_IL12_complex	1
IL18_IL18R1_IL18RAP_complex	1
IL18_TH1___extracellular_space	1
IL18_TH1___secreted_components	1
IL18R1_IL18RAP_complex	1
IL27	1
IL27_IL27RA_complex	1
IL7_IL7R_IL2RG_complex	0,5
IL9R_IL2RG_IL9_complex	1
IRAK1_IRAK4_complex	1
JAK1_JAK2_complex	1
JAK1_JAK2_TYK2_complex	1
JAK1_JAK3_complex	1
JAK1_TYK2_complex	1
JAK2_TYK2_complex	1
KRAS	1
LAT_phosphorylated	0
LGALS9	1
LGALS9_TIM3_complex	0
LTA_TH1___extracellular_space	0

LTA_TH1___secreted_components	0
LTA_TNFRSF14_complex	0
lyn	1
MAP2K1_phosphorylated	1
MAPK1_phosphorylated	1
MIF	1
MIF_CD74_complex	1
mtor	1
MYD88	1
NFKB1_RELA_complex_TH1___cytoplasm	1
NFKB1_RELA_complex_TH1___nucleus	1
NFKB1_RELA_NFKBIA_complex	1
NIK	1
PDCD1	1
PI3K_phosphorylated	1
PLCG1	1
PLXNB1	1
PRKCQ	1
proliferation_survival_TH1_phenotype	1
PTK2	1
Rac1	0
RAF1	1
RHOA	1
RPS6KA4	1
RUNX3	1
SEMA4_PLXNB1_complex	0,75
SEMA4A_TH1___extracellular_space	0,5
SEMA4A_TH1___secreted_components	0,5
SEMA4D	1
SH2D1A	0,5
SHC1_phosphorylated	1
SHIP1	0
SOS1	1
Src	1
STAT1	1
STAT1_STAT1_complex	1
STAT4	1
STAT4_STAT4_complex	1
tbx21	1
tbx21_phosphorylated	1
TNF	1
TNF_TNFRSF1B_complex	1
TNFA	1
TNFSF4	1
TNFSF4_TNFRSF4_complex	1
TRAF1_TRAF2_TRAF3_complex	1

TRAF2_TRAF5_complex	0
TRAF2_TRAF5_TRAF6_complex	1
TRAF6_phosphorylated	1
VAV1	0
VCAM1_ITGA4_ITGB7_complex	1
ZAP70	0

Supplementary Table 8. The list of additional differentially expressed biomolecules present in the multicellular model that we identified using literature search and omics data analysis. The first column contains the DEGs HGNC names. The second and fifth columns contain their corresponding Boolean values (after data discretization) observed in the cell-specific datasets and literature respectively.

DEG	Boolean value gene expression datasets	Adjusted p_value	logFC	Boolean value in literature	References
ACKR3	1	0,0001	5,13	1	33453247
AREG	1	1,00E-07	4,48		
BAX				1	12634940
CCL20				1	12695561
CCL3				1	15878203
CCL5	1	0,01	2,19		
CD40	1	0,002	2,29		
CD80	1	0,027	1,84		
CFLAR	1	0,002	0,8	1	12228167
COX2				1	32530555
CSF1				1	27036883
CXCL10	1	0,0004	6,02	1	32211348
CXCL11	1	0,0005	4,98		
CXCL12				1	12574387
CXCL13	1	0,0001	8,76	1	33292827
CXCL16				1	32211348
CXCL9	1	0,00004	5,13		
GNB1	1	0,005	0,67		
HBGEF				1	31068444
HES1	1	0,001	2,12		
HIF1A				1	11465705
HLA-DPB2	1	0,001	2,82		
HMGB1				1	35250993
ICAM1	1	0,044	0,68		
IL10				1	20001767
IL15	1	0,0001	2,69		
IL4				1	7492352
IRAK3	1	0,0001	1,51		
IRF5				1	26315890
ITCH	0	0,044	-0,6		

JAG1				1	28256007
LGALS9	1	0,008	0,73	1	32771893
LTA				1	29541795
MMP14				1	18567920
MMP3	1	0,014	3,55		
MMP9				1	18567920
NFKB1	1	0,002	0,89	1	8630106
NFKBIA	1	0,0002	0,98		
NFKBIE	1	0,01	0,79		
NOS2				1	9236674
RXRA	1	0,002	0,84		
SEMA4A				1	33605067
SOCS3	1	3,80E-07	5,11		
TNFAIP3	1	0,00005	2,36		
TRAF1	1	0,002	1,37		
TRAF6	0	0,006	-0,73		
VCAN	1	1,14E-06	5,2		
VEGFA	1	0,004	1,9		
ACVR2B	0	0,0004	-2,85		
CCL18				1	17350968
CCL20				1	12695561
COL4A3	1	0,008	2,8		
COX2				1	32530555
CXCL13	1	0,0001	8,76	1	33292827
CXCL16				1	32211348
FCGR2A	1	0,023	0,63	1	17521421
FN1	1	0,00007	4,74		
FZD5	1	0,001	1,83		
GAL				1	15616823
GNA13	1	0,00017	1,62		
HLA-DPB2	1	0,001	2,82		
IFNA	1	0,008	2,43		
IFNB				1	15878901
IL12				1	9756640
IL15	1	0,0001	2,69		
IL18				1	10562301
IL1B	1	0,004	2,17	1	15150426
IRF7	1	0,000018	2,44	1	22614743
ITCH	0	0,044	-0,6		
JAG1				1	28256007
MAP2K4				1	13130464
MAP2K7	1	0,048	0,72	1	13130464
MAP3K1	0	0,009	-0,72		
MAPK8	1	0,003	0,71		
MAPKAPK2	1	0,04	0,54		
MIF	1	0,00007	1,36		

MMP14				1	18567920
MMP3	1	0,014	3,55		
MMP9				1	18567920
NFKB1	1	0,002	0,89	1	8630106
NFKBIA	1	0,0002	0,98		
NFKBIE	1	0,01	0,79		
NLK	1	0,003	0,73		
OPN3	0	0,005	-0,87		
PDGFC				1	16508943
PLXNB2	1	0,00001	1,48		
PPRARG				1	22829690
PRKG1	1	0,003	2,17		
RAC1	1	0,033	0,74		
SEMA4D	1	0,0004	1,86		
SEMA7A				1	28109308
SMAD4	0	0,001	-0,81		
TCF7L2	1	0,019	1,97		
TNFAIP3	1	0,00005	2,36		
TRAF1	1	0,002	1,37		
ADAMTS4				1	22324945
ARRB2				1	21855149
BAK1				0	18177509
BMP6				1	14558086
BSG	1	0,002	0,59		
CCL21				1	21225692
CCL5				1	9756723
CCNB				1	16518573
CD40				1	10799861
CDKN1C	0	0,003	-1,63		
COL4A5	1	0,046	0,94		
CPNE4	1	0,003	3,09		
CSF1				1	27036883
CXCL1				1	7521808
CXCL2				1	33010041
CXCL3				1	29191223
CXCL9				1	15004722
DKK1				1	26785768
GAL				1	35023445
GAL				1	35023445
GAS6				1	34539648
HES1	1	0,003	0,64		
HLA-B				1	23259760
HLA-DRB1				1	
ICAM3	1	0,035	0,73		
IKK1				1	11160335
IL10				1	20001767

IL11				1	29327326
IL26				1	23055831
IL32				1	19248119
IL33				1	21431944
IL34				1	22264405
INHBB				1	26359667
IRF1				1	22401175
JAG1				1	32499639
JUNB	1	0,016	0,8	1	12905466
LGALS9				1	18050192
MAP3K7				1	17559674
MIR124A				0	21339227
MIR155				1	18383392
MIR203A				1	21279994
MIR221				1	25891943
MIR346				1	21611196
MIR34A				0	22161761
MMP9				1	3680518
NFKB1				1	12010604
NOTCH3				1	16307184
PDE4B	1	0,000975925	1,25		
PDIA3	1	0,0014184	0,82		
PLA2G2A	0	0,000480237	-1,67	1	21068383
PRL				1	27616146
RIPK3	0	0,028700766	-3,09		
RUNX1	0	0,000528477	-1,25		
SEMA3A	1	3,24E-12	1,76		
SEMA4D				1	25707877
STAT3				1	30477351
TGFB3	0	0,024324917	-1,62		
THBS1	0	4,40E-06	-0,73		
VCAM1	1	7,00E-05	1,71		
VEGFC	1	2,26E-08	1,18	1	11824968
CCL3	1	3,64E-18	6,85	1	32211348
CD28				1	12823856
EP300				1	31178673
GATA3				0	15981085
ICOS				1	11983910
IRF7				1	34432649
LAG3	1	1,46E-11	4,8		
NFAT				1	30538703
RBPJ				1	26604133
TCR				1	32115259
VCAM1	1	2,97E-15	7,01		

Supplementary Table 9. Calibrated state of the RA multicellular model

Nodes	Mean values
FOS	1
CDKN1A	0
CSF1R	1
CSK	1
CTF1	1
CYCS	0
CYLD	1
DUSP7	1
Dvl1	1
EDN1	1
EFNB1	1
EP300	1
FOXO3	0
GAB1	1
Grb2	1
HBEGF	1
IL1R1	1
IL4R	1
jag1	1
KRAS	1
LY96	1
map3k5	1
MCL1	1
mcl1	0
TIRAP	1
NRAS	0
PDE4B	1
PLCG1	1
PLXNB1	1
POU2F1	1
PRKCD	1
RPS6KA4	1
SOCS3	1
STAT6	1
TRAF2	1
vegfa	1
YWHAQ	0
ACKR3_CXCL12_complex	1
ACVR2A_ACVR2B_complex	0
ACVR2A_ACVR2B_INHBA_complex	0
ACVR2A_ACVR2B_INHBB_complex	0
ADAMTS4	1
ADAMTS9_rna	0
ADCY8	0
AGT	1

AGT_F2R_complex	1
AKT1_M1_macrophage___Cytoplasm	0
AKT1_M2_macrophage_nucleus	0
AKT1_phosphorylated	0
AKT1_TH1___cytoplasm	1
AKT2	1
AKT2_phosphorylated	1
AMAP1	1
angiogenesis_signal_phenotype	1
AP_1	1
AP_1_phosphorylated	1
APAF1	1
apoptosis_fibroblast_phenotype	0
apoptosis_M1_macrophage_phenotype	0
apoptosis_M2_macrophage_phenotype	1
apoptosis_TH1_phenotype	0
Apoptosome_complex	0
AREG_EGFR_complex	1
AREG_Fibroblast___Extracellular_Space	1
AREG_M1_macrophage___Secreted_components	1
ARHGEF12	1
ARHGEF2_Fibroblast___Cytoplasm	1
ARHGEF2_TH1___cytoplasm	1
ARI_ARII_bmp6_complex	0
ARRB2_Fibroblast___Cytoplasm	1
ARRB2_M1_macrophage___Cytoplasm	1
ARRB2_M2_macrophage__cytoplasm	1
ASC_M1_macrophage___Cytoplasm	1
ASC_M2_macrophage__cytoplasm	1
ASK1_M1_macrophage___Cytoplasm	1
ASK1_M2_macrophage__cytoplasm	1
BAD_Fibroblast___Mitochondrion	0
BAD_M1_macrophage__Mitochondria_membrane	0
BAD_M2_macrophage_mitochondrion_membrane	0
BAK1	0
BAX_Fibroblast___Mitochondrion	0
BAX_Fibroblast___Mitochondrion_active	0
BAX_M1_macrophage__Mitochondria_membrane	1
BAX_M2_macrophage_mitochondrion_membrane	1
BCL2_Fibroblast___Mitochondrion	1
BCL2_M1_macrophage__Mitochondria_membrane	1
BCL2_M1_macrophage__Mitochondria_membrane_active	0
BCL2_M2_macrophage_mitochondrion_membrane	1
BCL2_M2_macrophage_mitochondrion_membrane_active	0
Bcl2_rna_M1_macrophage___nucleus	1
Bcl2_rna_M2_macrophage_nucleus	1

BCL2A1_rna	1
BCL2L1_M1_macrophage__Mitochondria	1
BCL2L1_M1_macrophage__Mitochondria_active	0
BCL2L1_M2_macrophage__mitochondria	0
BCL2L1_rna	1
BCL2L11_Fibroblast___Cytoplasm	0
BCL2L11_Fibroblast___Cytoplasm_active	0
BCL2L11_TH1___cytoplasm	0
BCL3_rna_M1_macrophage___nucleus	1
BCL3_rna_M2_macrophage_nucleus	1
BID	0
biglycan_simple_molecule	1
Bim_M1_macrophage__Mitochondria	1
Bim_M2_macrophage__mitochondria	1
bmp6	1
BRAF_phosphorylated	0
BSG	1
BTK_rna	1
BTRC_rna	1
C_EBPb_phosphorylated	1
c_FOS_M1_macrophage___Cytoplasm	1
c_FOS_M1_macrophage___Cytoplasm_active	1
c_FOS_M1_macrophage___nucleus	1
c_JUN	1
c_JUN_phosphorylated_M1_macrophage___Cytoplasm	1
c_JUN_phosphorylated_M1_macrophage___nucleus	1
c_Myc_rna_M1_macrophage___nucleus	1
c_Myc_rna_M2_macrophage_nucleus	1
C5a_C5aR1_complex_M1_macrophage__Cytoplasmic_membrane_up	1
C5a_C5aR1_complex_M2_macrophage__cytoplasmic_membrane_up	1
c5a_M1_macrophage___Extracellular_Space	1
c5a_M2_macrophage__extracellular_space	1
CALCINEURIN	1
Casp1_M1_macrophage___Cytoplasm	1
Casp1_M2_macrophage__cytoplasm	1
CASP3_Fibroblast___Cytoplasm	0
CASP3_M1_macrophage___Cytoplasm	0
CASP3_M2_macrophage__cytoplasm	1
CASP7_M1_macrophage___Cytoplasm	0
CASP7_M2_macrophage__cytoplasm	1
CASP8_Fibroblast___Cytoplasm	0
CASP8_M1_macrophage___Cytoplasm	1
CASP8_M2_macrophage__cytoplasm	1
CASP9_Fibroblast___Cytoplasm	1
CASP9_M1_macrophage___Cytoplasm	0
CASP9_M2_macrophage__cytoplasm	0

CAV1_rna	1
CCL18_Fibroblast___Extracellular_Space	1
CCL18_M2_macrophage__secreted_components	1
CCL2_CCR2_complex_M1_macrophage__Cytoplasmic_membrane_up	1
CCL2_CCR2_complex_TH1___cytoplasmic_membrane_up	1
CCL2_Fibroblast___Extracellular_Space	1
CCL2_Fibroblast___secreted_components	1
CCL2_M1_macrophage___Extracellular_Space	1
CCL2_M1_macrophage___Secreted_components	1
CCL2_TH1___extracellular_space	1
CCL20_M1_macrophage___Secreted_components	1
CCL20_M2_macrophage__secreted_components	1
CCL21	1
CCL21_CCR7_complex_M1_macrophage__Cytoplasmic_membrane_up	1
CCL21_CCR7_complex_M2_macrophage__cytoplasmic_membrane_up	1
CCl21_M1_macrophage___Extracellular_Space	1
CCl21_M2_macrophage__extracellular_space	1
CCL3_M1_macrophage___Secreted_components	1
CCL3_TH1___secreted_components	1
CCL4_5_CCR5_complex	1
CCL4_M1_macrophage___Secreted_components	1
CCL4_TH1___extracellular_space	1
CCL4_TH1___secreted_components	1
CCL5_ACKR2_complex	1
CCL5_CCR5_complex	1
CCL5_Fibroblast___Extracellular_Space	1
CCL5_Fibroblast___secreted_components	1
CCL5_M1_macrophage___Secreted_components	1
CCL5_TH1___extracellular_space	1
CCL5_TH1___secreted_components	1
CCNA2	1
CCNB1_CDC2_complex	1
CCND1_rna	1
CCND2	1
CCR2_CCL2_complex	1
CCR5	1
CD28	1
CD28_CD86_complex	1
CD32a	1
CD32b_igG_complex	0
CD32b_rna	1
CD40_Fibroblast__Cytoplasmic_membrane_up	1
CD40_M1_macrophage__Cytoplasmic_membrane_down	1
CD40LG_CD40_complex	1
CD40LG_ITGAM_ITGB2_complex	1
CD40LG_ITGB1_ITGA1_complex_M1_macrophage__Cytoplasmic_membrane_up	1

CD40LG_ITGB1_ITGA1_complex_M2_macrophage__cytoplasmic_membrane_up	1
CD40LG_M1_macrophage__Cytoplasmic_membrane_down	1
CD40LG_M2_macrophage__cytoplasmic_membrane_down	1
CD40LG_TH1___cytoplasmic_membrane_down	1
CD80	1
CD84_CD84_complex	1
CD84_Fibroblast__Cytoplasmic_membrane_down	1
CD84_M1_macrophage__Cytoplasmic_membrane_down	1
CD84_M2_macrophage__cytoplasmic_membrane_down	1
CD84_TH1___cytoplasmic_membrane_up	1
CD86_M1_macrophage__Cytoplasmic_membrane_down	1
CD86_M2_macrophage__cytoplasmic_membrane_down	1
CDC25B_C	0
Cell_chemotaxis_migration_fibroblast_phenotype	1
Cell_chemotaxis_migration_TH1_phenotype	1
CFLAR_Fibroblast___Cytoplasm	1
CFLAR_Fibroblast___Cytoplasm_active	1
cFLIP_M1_macrophage___Cytoplasm	1
cFLIP_M1_macrophage___Cytoplasm_active	1
cFLIP_M2_macrophage__cytoplasm	1
cFLIP_M2_macrophage__cytoplasm_active	1
cGAS	1
CHUK_phosphorylated	1
cIAP1	1
cMyc	1
cMyc_phosphorylated_M2_macrophage__cytoplasm	1
cMyc_phosphorylated_M2_macrophage_nucleus	1
col4a3_Fibroblast___Extracellular_Space	1
COL4A3_ITGA4_ITGB7_complex	1
col4a3_M2_macrophage__secreted_components	1
col4a3_TH1___extracellular_space	1
col4a4_Fibroblast___secreted_components	1
col4a4_M1_macrophage___Extracellular_Space	1
col4a4_M2_macrophage__extracellular_space	1
COL4A5	1
col4a5_Fibroblast___Extracellular_Space	1
col4a5_M1_macrophage___Extracellular_Space	1
col4a5_M2_macrophage__extracellular_space	1
COMP	1
COX2_rna_M1_macrophage___nucleus	1
COX2_rna_M2_macrophage_nucleus	1
CPNE3_rna	1
CREB1_phosphorylated_Fibroblast___nucleus	1
CREB1_phosphorylated_M2_macrophage_nucleus	0
CREB1_phosphorylated_TH1___nucleus	1
CRKL_phosphorylated_Fibroblast___Cytoplasm	1

CRKL_phosphorylated_M1_macrophage___Cytoplasm	1
CSF1_Fibroblast___secreted_components	1
CSF1_M2_macrophage__extracellular_space	1
CSF1_M2_macrophage__secreted_components	1
CSF1_rna	1
CSF1R_CSF1_complex	1
CSF2_Fibroblast___Extracellular_Space	1
CSF2_Fibroblast___secreted_components	1
CSF2_M1_macrophage___Extracellular_Space	1
CSF2_M1_macrophage___Secreted_components	1
CSF2RA_CSF2RB_complex_Fibroblast__Cytoplasmic_membrane_up	1
CSF2RA_CSF2RB_complex_M1_macrophage__Cytoplasmic_membrane_up	1
CSF2RA_CSF2RB_CSF2_complex	1
CSFR1R_IL34_complex	1
CSL	1
CTNNB_CK1A_AXIN_GSK3B_APC_complex	0
CTNNB1_Fibroblast___nucleus	0
CTNNB1_M2_macrophage_nucleus	1
CTNNB1_TCF_LEF_complex	1
CTSK_rna	1
CXCL1_CXCR1_complex	1
CXCL1_Fibroblast___secreted_components	1
CXCL1_M1_macrophage___Extracellular_Space	1
CXCL1_M1_macrophage___Secreted_components	1
CXCL10_CXCR3_complex_Fibroblast__Cytoplasmic_membrane_up	1
CXCL10_CXCR3_complex_TH1___cytoplasmic_membrane_up	1
CXCL10_Fibroblast___Extracellular_Space	1
CXCL10_Fibroblast___secreted_components	1
CXCL10_M1_macrophage___Secreted_components	1
CXCL10_TH1___extracellular_space	1
CXCL10_TH1___secreted_components	1
CXCL11	1
CXCL12	1
CXCL13_ACKR4_complex_M1_macrophage__Cytoplasmic_membrane_up	1
CXCL13_ACKR4_complex_M2_macrophage__cytoplasmic_membrane_up	1
CXCL13_CXCR3_complex	1
CXCL13_Fibroblast___Extracellular_Space	1
CXCL13_M1_macrophage___Extracellular_Space	1
CXCL13_M2_macrophage__extracellular_space	1
CXCL13_TH1___secreted_components	1
CXCL16_CXCR6_complex	1
CXCL16_M1_macrophage___Secreted_components	1
CXCL16_M2_macrophage__secreted_components	1
CXCL16_TH1___extracellular_space	1

CXCL2	1
CXCL3	1
CXCL8_Fibroblast___Extracellular_Space	1
CXCL8_Fibroblast___secreted_components	1
CXCL9_Fibroblast___secreted_components	1
CXCL9_M1_macrophage___Secreted_components	1
CXCR1	1
CXCR1_IL8_complex_M1_macrophage__Cytoplasmic_membrane_up	1
CXCR1_IL8_complex_M2_macrophage__cytoplasmic_membrane_up	1
CXCR2_CXCL8_complex	1
CXCR3	1
CypA	1
DAG_simple_molecule	1
DAXX_Fibroblast___Cytoplasm	1
DAXX_M1_macrophage___Cytoplasm	1
DAXX_M2_macrophage__cytoplasm	1
DAXX_TH1___cytoplasm	1
DKK1	1
DNA_simple_molecule	1
DOCK2	1
DOCK2_CRKL_complex	1
dsRNA_simple_molecule	1
DUSP1_M1_macrophage___Cytoplasm	1
DUSP1_M2_macrophage__cytoplasm	1
DVL1_phosphorylated	1
DYNLRB1	1
ECSIT	1
EDA_EDA2R_complex	1
EDA_Fibroblast___Extracellular_Space	1
EDA_M1_macrophage___Secreted_components	1
EDA_M2_macrophage__secreted_components	1
EFNB1_EPHB1_complex	1
efnb1_rna	1
EGF	1
EGF_EGFR_complex	1
EGFR	1
ELK1_phosphorylated	1
EOMES	1
EPHB2	1
ERK1_phosphorylated_M1_macrophage___Cytoplasm	1
ERK1_phosphorylated_M1_macrophage___nucleus	1
ERK1_phosphorylated_M2_macrophage__cytoplasm	1
ERK1_phosphorylated_M2_macrophage_nucleus	1
FADD_Fibroblast___Cytoplasm	1
FADD_M1_macrophage___Cytoplasm	1
FADD_M2_macrophage__cytoplasm	1

FAS_FASL_complex	1
FAS_M1_macrophage__Cytoplasmic_membrane_up	1
FAS_M2_macrophage__cytoplasmic_membrane_up	1
FASL_FAS_complex_M1_macrophage__Cytoplasmic_membrane_up	1
FASL_FAS_complex_M2_macrophage__cytoplasmic_membrane_up	1
FASL_M1_macrophage___Extracellular_Space	1
FASL_M1_macrophage___Secreted_components	1
FASL_M2_macrophage__extracellular_space	1
FASL_M2_macrophage__secreted_components	1
FASLG_FAS_complex	1
FASLG_Fibroblast___Extracellular_Space	1
FASLG_TH1___extracellular_space	1
FASLG_TH1___secreted_components	1
FGF1	1
FGF1_FGFR4_complex	1
FGR	1
FN1_Fibroblast___Extracellular_Space	0
FN1_Fibroblast___secreted_components	0
FN1_ITGAV_complex	0
FN1_M2_macrophage__secreted_components	1
FOXO_M1_macrophage___Cytoplasm	1
FOXO_M1_macrophage___nucleus	1
FOXO1_Fibroblast___nucleus	0
FOXO1_M2_macrophage_nucleus	1
FZD1_LRP5_complex	1
FZD5_LRP5_wnt5a_complex	1
GAB2_phosphorylated_Fibroblast___Cytoplasm	1
GAB2_phosphorylated_M2_macrophage__cytoplasm	1
gal_Fibroblast___secreted_components	1
GAL_GALR2_complex_M1_macrophage__Cytoplasmic_membrane_up	1
GAL_GALR2_complex_M2_macrophage__cytoplasmic_membrane_up	1
gal_M1_macrophage___Extracellular_Space	1
gal_M2_macrophage__extracellular_space	1
GAL_rna	1
gamma_secretase_complex	1
gamma_secretase_complex_complex	1
GAS6_Fibroblast___secreted_components	1
GAS6_M2_macrophage__extracellular_space	1
GAS6_MERTK_complex	1
gas6_mertk_complex	1
GAS6_TH1___extracellular_space	1
gata3_rna	0
GNA12_GNA13_complex_M1_macrophage___Cytoplasm	1
GNA12_GNA13_complex_M2_macrophage__cytoplasm	1
GNA12_GNA13_complex_TH1___cytoplasm	1
GNAI_GNB_GNG_complex	1

GNAI_TH1___cytoplasm	1
GNAI_TH1___cytoplasm_active	1
GNAI3_Fibroblast___Cytoplasm	1
GNAI3_M1_macrophage___Cytoplasm	1
GNAI3_M2_macrophage__cytoplasm	1
GNB_GNG_complex_M1_macrophage___Cytoplasm	1
GNB_GNG_complex_M2_macrophage__cytoplasm	1
GNB_GNG_complex_TH1___cytoplasm	1
GNB_GNG_GNAI3_complex_M1_macrophage___Cytoplasm	1
GNB_GNG_GNAI3_complex_M2_macrophage__cytoplasm	1
GRB2_Fibroblast___Cytoplasm	1
GRB2_TH1___cytoplasm	1
GSK3B	0
GSK3b_APC_AXIN1_CK1A_complex	1
GSK3b_APC_AXIN1_CTNNB1_CK1A_complex	1
HBGEF_EGFR_complex	1
HBGEF_M1_macrophage___Secreted_components	1
HBGEF_M2_macrophage__secreted_components	1
HCK	1
HES1_rna	1
hes1_rna	1
HEY1_rna	1
HIF1_complex	1
Hif1a	1
Hif1b	1
HLA_B	1
HLA_B_LILRB1_complex_M1_macrophage__Cytoplasmic_membrane_up	1
HLA_B_LILRB1_complex_M2_macrophage__cytoplasmic_membrane_up	1
HLA_DP_DQ_DR_LAG3_complex	1
HLA_DP_DQ_DR_M1_macrophage__Cytoplasmic_membrane_down	1
HLA_DP_DQ_DR_M2_macrophage__cytoplasmic_membrane_down	1
HLA_DP_DQ_DR_TCR_CD3_complex	1
HLA_DRB1_rna	1
HMGB1	1
HOMODIMER_space_STAT1	1
HOMODIMER_space_STAT3	1
HRAS_M1_macrophage___Cytoplasm	1
HRAS_M2_macrophage__cytoplasm	1
HSPA5	1
ICAM1_Fibroblast__Cytoplasmic_membrane_down	1
ICAM1_ITGB2_ITGAL_complex	1
ICAM1_M1_macrophage__Cytoplasmic_membrane_down	1
ICAM1_TH1___cytoplasmic_membrane_down	1
icos	1
ICOSLG_Fibroblast__Cytoplasmic_membrane_down	1

icoslg_icos_complex	1
ICOSLG_M1_macrophage__Cytoplasmic_membrane_down	1
ICOSLG_M2_macrophage__cytoplasmic_membrane_down	1
IFNa_M1_macrophage___Extracellular_Space	1
IFNa_M1_macrophage___Secreted_components	1
IFNa_rna	1
IFNa1	1
IFNa1_B1	1
IFNA1_B1_IFNAR1_R2_complex	1
IFNAR1_IFNAR2_complex	1
IFNAR1_IFNAR2_IFNa_complex	1
IFNAR1_IFNAR2_IFNb_complex	1
IFNb_M1_macrophage___Extracellular_Space	1
IFNb_M1_macrophage___Secreted_components	1
IFNb_rna	1
IFNb1	1
IFNB1_rna	1
IFNE	1
IFNE_IFNAR1_IFNAR2_complex	1
IFNg_Fibroblast___Extracellular_Space	1
IFNG_IFNGR1_IFNGR2_complex	1
IFNG_IFNGR1_R2_complex	1
IFNg_M1_macrophage___Extracellular_Space	1
IFNg_M1_macrophage___Secreted_components	1
IFNg_rna	1
IFNg_TH1___extracellular_space	1
IFNg_TH1___secreted_components	1
IFNGR1_IFNGR2_complex_M1_macrophage__Cytoplasmic_membrane_up	1
IFNGR1_IFNGR2_complex_TH1___cytoplasmic_membrane_up	1
IFNGR1_IFNGR2_IFNg_complex	1
IFNGR1_R2_complex	1
IGF1	0
IGF1_IGF1R_complex	0
igG	0
IKBA_NFKB1_RELA_complex	1
IKBKB_phosphorylated	1
IKK_complex_Fibroblast___Cytoplasm	1
IKK_complex_M1_macrophage___Cytoplasm	1
IKK_complex_M2_macrophage__cytoplasm	1
IKK_complex_TH1___cytoplasm	1
IKK1_IKK2_complex_Fibroblast___Cytoplasm	1
IKK1_IKK2_complex_M1_macrophage___Cytoplasm	1
IKK1_IKK2_complex_M2_macrophage__cytoplasm	1
IKK1_phosphorylated_Fibroblast___Cytoplasm	1
IKK1_phosphorylated_M1_macrophage___Cytoplasm	1
IKK1_phosphorylated_M2_macrophage__cytoplasm	1

IKK2_phosphorylated_Fibroblast___Cytoplasm	1
IKK2_phosphorylated_M1_macrophage___Cytoplasm	1
IKK2_phosphorylated_M2_macrophage__cytoplasm	1
IKKE_TBK1_complex	1
IKKE_TBK1_TRAF3_complex	0
IL1_IL1R_complex	1
IL10_Fibroblast___Extracellular_Space	1
IL10_IL10RA_IL10RB_complex	1
IL10_M2_macrophage__extracellular_space	1
IL10_M2_macrophage__secreted_components	1
IL10_rna	1
IL10_TH1___extracellular_space	1
IL10R1_IL10R2_complex	1
IL10R1_IL10R2_IL10_complex	1
IL10RA_IL10RB_complex_Fibroblast__Cytoplasmic_membrane_up	1
IL10RA_IL10RB_complex_TH1___cytoplasmic_membrane_up	1
IL10RA_IL10RB_IL10_complex	1
IL11	1
IL11_IL11Ra_IL6ST_complex	1
IL11Ra_IL6ST_IL11_complex	1
IL12_M1_macrophage___Extracellular_Space	1
IL12_M1_macrophage___Secreted_components	1
IL12_rna	1
IL12_TH1___extracellular_space	1
IL12_TH1___secreted_components	1
IL12A	1
IL12B	1
IL12RB_complex	1
IL12RB_IL12_complex	1
IL12Rb1_IL12Rb2_complex	1
IL12Rb1_IL12Rb2_IL12_complex	1
IL15_M1_macrophage___Secreted_components	1
IL15_M2_macrophage__secreted_components	1
IL17A_Fibroblast___Extracellular_Space	1
IL17A_Fibroblast___secreted_components	1
IL17A_IL17RA_complex	1
IL17F	1
IL17Ra_IL17Rc_IL17F_complex	1
IL18_Fibroblast___Extracellular_Space	1
IL18_Fibroblast___secreted_components	1
IL18_IL18R_complex	1
IL18_IL18R1_complex	1
IL18_IL18R1_IL18RAP_complex	1
IL18_M1_macrophage___Cytoplasm	1
IL18_M1_macrophage___Extracellular_Space	1
IL18_M1_macrophage___Secreted_components	1

IL18_M2_macrophage__cytoplasm	1
IL18_M2_macrophage__cytoplasm_active	1
IL18_TH1___extracellular_space	1
IL18_TH1___secreted_components	1
IL18R1_IL18RAP_complex	1
IL1A	1
IL1A_IL1R1_complex	1
IL1B_Fibroblast___Extracellular_Space	1
IL1B_Fibroblast___secreted_components	1
IL1B_IL1R1_complex	1
IL1B_M1_macrophage___Cytoplasm	1
IL1B_M1_macrophage___Extracellular_Space	1
IL1B_M1_macrophage___Secreted_components	1
IL1B_M2_macrophage__cytoplasm	1
IL1RN_rna	1
IL2	1
IL23_M1_macrophage___Extracellular_Space	1
IL23_M1_macrophage___Secreted_components	1
IL23_M2_macrophage__extracellular_space	1
IL23_M2_macrophage__secreted_components	1
IL23R_IL12RB1_complex_M1_macrophage__Cytoplasmic_membrane_up	1
IL23R_IL12RB1_complex_M2_macrophage__cytoplasmic_membrane_up	1
IL23R_IL12RB1_IL23_complex_M1_macrophage__Cytoplasmic_membrane_up	1
IL23R_IL12RB1_IL23_complex_M2_macrophage__cytoplasmic_membrane_up	1
IL26	1
IL27	1
IL27_IL27RA_complex	1
IL32_rna	1
IL33_rna	1
IL34_Fibroblast___secreted_components	1
IL34_M2_macrophage__extracellular_space	1
IL4	1
IL4_IL4Ra_complex	1
IL4_rna	1
IL6_Fibroblast___Extracellular_Space	1
IL6_Fibroblast___secreted_components	1
IL6_IL6R_complex	1
IL6_IL6R_IL6ST_complex	1
IL6_IL6ST_complex	1
IL6_M1_macrophage___Extracellular_Space	1
IL6_M1_macrophage___Secreted_components	1
IL7_Fibroblast___secreted_components	1
IL7_IL7R_IL2RG_complex	1

IL7_TH1___extracellular_space	1
IL7R_IL2RG_complex	1
IL8_M1_macrophage___Extracellular_Space	1
IL8_M1_macrophage___Secreted_components	1
IL8_M2_macrophage__extracellular_space	1
IL8_M2_macrophage__secreted_components	1
IL9R_IL2RG_IL9_complex	1
immune_complex_CD16a_complex	1
immune_complex_CD32a_complex	1
immune_complex_CD32B_complex	1
immune_complex_CD64_complex	1
immune_complex_complex	1
inflammation_signal_phenotype	1
INHBA	1
INHBB_Fibroblast___secreted_components	1
INHBB_M1_macrophage___Extracellular_Space	1
INPP5A	1
IP3_simple_molecule	1
IRAK1_Fibroblast___Cytoplasm	1
IRAK1_Fibroblast___Cytoplasm_active	1
IRAK1_IRAK4_complex	1
IRAK1_IRAK4_complex_M1_macrophage___Cytoplasm	1
IRAK1_IRAK4_complex_TH1___cytoplasm	1
IRAK1_M1_macrophage___Cytoplasm	1
IRAK3	1
IRAK4_Fibroblast___Cytoplasm	1
IRAK4_Fibroblast___Cytoplasm_active	1
IRAK4_phosphorylated	1
IRF1_Fibroblast___Cytoplasm	1
IRF1_Fibroblast___nucleus	1
IRF3_phosphorylated_Fibroblast___nucleus	1
IRF3_phosphorylated_M1_macrophage___nucleus	1
IRF5_Fibroblast___Cytoplasm	1
IRF5_Fibroblast___nucleus	1
IRF5_ubiquitinated	1
IRF7_M1_macrophage___nucleus	1
IRF7_M2_macrophage_nucleus	1
IRF7_TH1___cytoplasm	1
IRF7_TH1___cytoplasm_active	1
IRF9_Fibroblast___Cytoplasm	1
IRF9_M1_macrophage___Cytoplasm	1
ISGF3_complex	1
ITCH_phosphorylated_M1_macrophage___Cytoplasm	0
ITCH_phosphorylated_M2_macrophage__cytoplasm	0
ITGA4_ITGB1_col4a3_complex	1
ITGA4_ITGB1_complex	1

ITGA4_ITGB1_sema7a_complex	1
ITGA4_ITGB7_complex	1
ITGA5_ITGB1_col4a5_complex	1
ITGA5_ITGB1_complex	1
ITGAL_ITGB2_complex	1
ITGAM_ITGB2_complex	1
ITGB1_ITGA1_col4a_complex_M1_macrophage__Cytoplasmic_membrane_up	1
ITGB1_ITGA1_col4a_complex_M2_macrophage__cytoplasmic_membrane_up	1
ITGB1_ITGA1_complex_M1_macrophage__Cytoplasmic_membrane_up	1
ITGB1_ITGA1_complex_M2_macrophage__cytoplasmic_membrane_up	1
JAG1_M1_macrophage__Cytoplasmic_membrane_down	1
JAG1_M2_macrophage__cytoplasmic_membrane_down	1
JAG1_NOTCH1_complex	1
JAG1_NOTCH3_complex	1
JAG1_rna	1
JAK1_Fibroblast___Cytoplasm	0
JAK1_JAK2_complex_M1_macrophage___Cytoplasm	1
JAK1_JAK2_complex_TH1___cytoplasm	1
JAK1_JAK2_TYK2_complex	1
JAK1_JAK3_complex	1
JAK1_M1_macrophage___Cytoplasm	1
JAK1_M2_macrophage__cytoplasm	1
JAK1_TYK2_complex_M1_macrophage___Cytoplasm	1
JAK1_TYK2_complex_M2_macrophage__cytoplasm	1
JAK1_TYK2_complex_TH1___cytoplasm	1
JAK2_Fibroblast___Cytoplasm	0
JAK2_M1_macrophage___Cytoplasm	1
JAK2_M2_macrophage__cytoplasm	1
JAK2_TYK2_complex_M1_macrophage___Cytoplasm	1
JAK2_TYK2_complex_TH1___cytoplasm	1
JAK3	0
JNK1_phosphorylated_M1_macrophage___Cytoplasm	1
JNK1_phosphorylated_M1_macrophage___nucleus	1
JNK1_phosphorylated_M2_macrophage_nucleus	1
JUN_phosphorylated	1
JUNB	1
klf4	1
LAG3	1
LAT_phosphorylated	0
LBP_CD14_complex	1
LCK_phosphorylated	0
LGALS9_Fibroblast___secreted_components	1
LGALS9_M1_macrophage___Secreted_components	1
LGALS9_TH1___extracellular_space	1
LGALS9_TIM3_complex	0

LIFR_IL6ST_CTF1_complex	1
LTA_Fibroblast___secreted_components	1
LTA_M1_macrophage___Secreted_components	1
LTA_TH1___extracellular_space	1
LTA_TH1___secreted_components	1
LTA_TNFRSF14_complex	1
LTBP1	0
LY96_TLR2_4_complex	1
lyn	1
maf_rna	0
MAML1	1
MAP2K1_phosphorylated_Fibroblast___Cytoplasm	1
MAP2K1_phosphorylated_TH1___cytoplasm	1
MAP2K3_phosphorylated	1
MAP2K4_phosphorylated	1
MAP2K6_phosphorylated	1
MAP2K7_phosphorylated	1
MAP3K1_phosphorylated	1
MAP3K14_phosphorylated	1
MAP3K2_3_4	1
MAP3K5_phosphorylated	1
MAP3K7_phosphorylated	1
MAP3K7_rna	1
MAP3K8_phosphorylated	1
MAP4K4_phosphorylated	1
MAPK1_empty	0
MAPK1_phosphorylated_Fibroblast___Cytoplasm	0
MAPK1_phosphorylated_TH1___cytoplasm	1
MAPK14_phosphorylated	1
MAPK3_complex	1
MAPK3_phosphorylated	1
MAPK8	1
MAPK8_phosphorylated	1
MAPK9_phosphorylated	1
MAPKAPK2_phosphorylated	1
Matrix_degradation_signal_phenotype	1
Mcl1_rna_M1_macrophage___nucleus	1
Mcl1_rna_M2_macrophage_nucleus	0
MDM2	1
MDM2_phosphorylated_Fibroblast___Cytoplasm	1
MDM2_phosphorylated_M1_macrophage___Cytoplasm	0
MDM2_phosphorylated_M2_macrophage__cytoplasm	0
MEK1_phosphorylated_M1_macrophage___Cytoplasm	1
MEK1_phosphorylated_M2_macrophage__cytoplasm	1
MEK2_phosphorylated_M1_macrophage___Cytoplasm	1
MEK2_phosphorylated_M2_macrophage__cytoplasm	1

MEKK1_M1_macrophage___Cytoplasm	1
MEKK1_M2_macrophage__cytoplasm	0
MIF_CD74_complex	1
MIF_CXCR4_complex	1
MIF_Fibroblast___Extracellular_Space	1
MIF_Fibroblast___secreted_components	1
MIF_M2_macrophage__secreted_components	1
MIF_TH1___extracellular_space	1
MIF_TH1___secreted_components	1
MIR10a_rna	0
MIR124A_rna	0
MIR146A_rna	0
MIR155_rna	1
MIR192_rna	0
MIR203A_rna	1
MIR221_rna	1
MIR338_5P_rna	1
MIR346_rna	1
MIR34A_rna	0
MIR451A_rna	0
MIR650_rna	0
MK2_phosphorylated_M1_macrophage___nucleus	1
MK2_phosphorylated_M2_macrophage_nucleus	1
MKK3_phosphorylated_M1_macrophage___Cytoplasm	1
MKK3_phosphorylated_M2_macrophage__cytoplasm	1
MKK4_phosphorylated_M1_macrophage___Cytoplasm	1
MKK4_phosphorylated_M2_macrophage__cytoplasm	1
MKK6_phosphorylated_M1_macrophage___Cytoplasm	1
MKK6_phosphorylated_M2_macrophage__cytoplasm	1
MKK7_phosphorylated_M1_macrophage___Cytoplasm	1
MKK7_phosphorylated_M2_macrophage__cytoplasm	1
MMP1	1
MMP13	1
MMP14_M1_macrophage___Secreted_components	1
MMP14_M2_macrophage__secreted_components	1
MMP3_Fibroblast___secreted_components	1
MMP3_M1_macrophage___Secreted_components	1
MMP3_M2_macrophage__secreted_components	1
MMP9_Fibroblast___secreted_components	1
MMP9_M1_macrophage___Secreted_components	1
MMP9_M2_macrophage__secreted_components	1
MSK1_phosphorylated	0
mtor	1
MYD88_Fibroblast___Cytoplasm	1
MYD88_M1_macrophage___Cytoplasm	1
MYD88_TH1___cytoplasm	1

MYD88_TIRAP_TOLLIP_complex	1
NCID_Fibroblast___nucleus	1
NCID_M1_macrophage___Cytoplasm	1
ncid_rbpj_snw1_complex	1
NECTIN3_PTPRC_NECTIN1_complex	1
NFAT	1
NFAT5	1
NFAT5_phosphorylated_Fibroblast___nucleus	1
NFAT5_phosphorylated_M2_macrophage_nucleus	1
NFKB_complex	1
NFKB_N_complex	1
NFKB1_Fibroblast___Cytoplasm	1
NFKB1_M1_macrophage___Cytoplasm	1
NFKB1_M2_macrophage__cytoplasm	1
NFKB1_MAP3K8_complex	1
NFKB1_RELA_complex_TH1___cytoplasm	1
NFKB1_RELA_complex_TH1___nucleus	1
NFKB1_RELA_NFKBIA_complex	1
NFKB1_TPL2_complex_M1_macrophage___Cytoplasm	1
NFKB1_TPL2_complex_M2_macrophage__cytoplasm	1
NFKB2_RELB_complex	1
NFKBIA_phosphorylated_Fibroblast___Cytoplasm	1
NFKBIA_phosphorylated_M1_macrophage___Cytoplasm	1
NFKBIA_phosphorylated_M2_macrophage__cytoplasm	1
NFKBIA_phosphorylated_TH1___cytoplasm	1
NFKBIA_RELA_NFKB1_complex_M1_macrophage___Cytoplasm	1
NFKBIA_RELA_NFKB1_complex_M2_macrophage__cytoplasm	1
NFKBIA_rna	1
NFKBIE_Fibroblast___Cytoplasm	1
NFKBIE_M1_macrophage___Cytoplasm	1
NFKBIE_M2_macrophage__cytoplasm	1
ngef	1
NICD_CSL_SKIP_MAML1_ep300_complex	1
NICD_EP300_SKIP_CSL_MAML1_complex	1
NICD_M1_macrophage___nucleus	1
NICD_TH1___nucleus	1
NIK_M1_macrophage___Cytoplasm	1
NIK_TH1___cytoplasm	1
NLK_phosphorylated	1
NLRP3_INFLAMMASOME_complex_M1_macrophage___Cytoplasm	1
NLRP3_INFLAMMASOME_complex_M2_macrophage__cytoplasm	1
NLRP3_M1_macrophage___Cytoplasm	1
NLRP3_M2_macrophage__cytoplasm	1
NOD2	0
NOS2	1
NOS2_phosphorylated	0

NOTCH1	1
notch1_JAG1_complex	1
notch3	1
OPN_M1_macrophage___Cytoplasm	0
OPN_M2_macrophage__cytoplasm	0
osteoclastogenesis_M1_macrophage_phenotype	1
osteoclastogenesis_signal_phenotype	1
p15_rna_M1_macrophage___nucleus	0
p15_rna_M2_macrophage_nucleus	1
p21_rna	1
p300_SP1_complex_M1_macrophage___nucleus	0
p300_SP1_complex_M2_macrophage_nucleus	0
p38_MAP_KINASE_phosphorylated_M1_macrophage___Cytoplasm	0
p38_MAP_KINASE_phosphorylated_M1_macrophage___nucleus	0
p38_MAP_KINASE_phosphorylated_M2_macrophage__cytoplasm	0
p38MAPK_empty	1
p38MAPK_phosphorylated	0
p53	1
p53_phosphorylated_M1_macrophage___Cytoplasm	1
p53_phosphorylated_M2_macrophage__cytoplasm	1
p53_phosphorylated_M2_macrophage_nucleus	1
PDCD1	1
PDGFC_Fibroblast___Extracellular_Space	1
PDGFC_M2_macrophage__secreted_components	1
PDGFC_PDGFRB_complex	1
PDIA3_HLA_A_B2M_complex	1
PI3_4_5_P__3_simple_molecule	0
PI3K_M1_macrophage___Cytoplasm	0
PI3K_M2_macrophage__cytoplasm	0
PI3K_phosphorylated	1
PI4_5_P__2_simple_molecule	1
PIK3AP1_phosphorylated_M1_macrophage___Cytoplasm	0
PIK3AP1_phosphorylated_M2_macrophage__cytoplasm	0
PIK3R5_phosphorylated	1
PIP2_simple_molecule	1
PITPNM3_CCL18_complex	1
PLA2G2A_phosphorylated	1
PLCG2	1
PLXNA1	1
PLXNA1_sema3A_complex	1
PLXNB1_MET_complex	1
PLXNB1_MET_sema4a_complex	1
PLXNB2_MET_complex	1
PMAIP1_Fibroblast___Cytoplasm	1
PMAIP1_Fibroblast___Cytoplasm_active	1
PP2A	0

PP4	1
PPARg_rna	1
PRKACA	1
Prkcd_M1_macrophage___Cytoplasm	1
Prkcd_M2_macrophage__cytoplasm	1
PRKCQ_Fibroblast___Cytoplasm	1
PRKCQ_M1_macrophage___Cytoplasm	1
PRKCQ_M2_macrophage__cytoplasm	1
PRKCQ_TH1___cytoplasm	1
PRKG1_Fibroblast___Cytoplasm	1
PRKG1_M1_macrophage___Cytoplasm	1
PRKG1_M2_macrophage__cytoplasm	1
PRL_Fibroblast___secreted_components	1
PRL_M2_macrophage__extracellular_space	1
PRL_PRLR_complex	1
proliferation_survival_fibroblast_phenotype	1
proliferation_survival_M1_macrophage_phenotype	1
proliferation_survival_M2_macrophage_phenotype	0
proliferation_survival_TH1_phenotype	1
PTEN	1
PTGS2_rna	1
PTK2_M1_macrophage___Cytoplasm	1
PTK2_M2_macrophage__cytoplasm	1
PTK2_TH1___cytoplasm	1
PTK2B_phosphorylated	1
PTPN11_phosphorylated	1
PTPN6_Fibroblast___Cytoplasm	1
PTPN6_M1_macrophage___Cytoplasm	1
PTPN6_M2_macrophage__cytoplasm	1
pyk2_phosphorylated	1
RAB5A	1
RAC1_2	1
Rac1_M1_macrophage___Cytoplasm	1
Rac1_M2_macrophage__cytoplasm	1
Rac1_TH1___cytoplasm	0
RAF1_Fibroblast___Cytoplasm	1
RAF1_M1_macrophage___Cytoplasm	1
RAF1_M2_macrophage__cytoplasm	1
RAF1_TH1___cytoplasm	1
RANK_RANKL_complex	1
rasa1	1
RBL1_E2F4_DP1_complex	0
rbpj	1
RDX	1
RELA_NFKB1_complex_M1_macrophage___Cytoplasm	1
RELA_NFKB1_complex_M1_macrophage___nucleus	1

RELA_NFKB1_complex_M2_macrophage__cytoplasm	1
RELA_NFKB1_complex_M2_macrophage_nucleus	1
RELA_NFKB1_NFKBIE_complex_Fibroblast___Cytoplasm	1
RELA_NFKB1_NFKBIE_complex_M1_macrophage___Cytoplasm	1
RELA_NFKB1_NFKBIE_complex_M2_macrophage__cytoplasm	1
RHOA_Fibroblast___Cytoplasm	0
RHOA_M1_macrophage___Cytoplasm	0
RHOA_M2_macrophage__cytoplasm	0
RHOA_TH1___cytoplasm	1
RIPK1	1
RIPK1_TRAF6_complex	1
RIPK3	0
RPS6KB1_phosphorylated	1
RUNX1	0
RUNX3	1
RXRa_NUR77_complex_M1_macrophage___Cytoplasm	1
RXRa_NUR77_complex_M2_macrophage__cytoplasm	1
SARA_M1_macrophage___Cytoplasm	0
SARA_M2_macrophage__cytoplasm	0
SARM1	0
sema3A	1
SEMA4_PLXNB1_complex	1
SEMA4_PLXNB2_MET_complex	1
SEMA4A_Fibroblast___Extracellular_Space	1
SEMA4A_M1_macrophage___Secreted_components	1
SEMA4A_M2_macrophage__extracellular_space	1
SEMA4A_M2_macrophage__secreted_components	1
SEMA4A_TH1___extracellular_space	1
SEMA4A_TH1___secreted_components	1
SEMA4D_Fibroblast___secreted_components	1
SEMA4D_M2_macrophage__extracellular_space	1
SEMA4D_TH1___extracellular_space	1
sema7a_Fibroblast___Extracellular_Space	1
sema7a_M2_macrophage__secreted_components	1
SERPINE1_rna	1
SH2D1A_Fibroblast___Cytoplasm	1
SH2D1A_M2_macrophage__cytoplasm	1
SH2D1A_TH1___cytoplasm	1
Shc_phosphorylated	1
SHC1_phosphorylated	1
SHC2_phosphorylated	1
SHIP1_M2_macrophage__cytoplasm	1
SHIP1_TH1___cytoplasm	0
SHP2_GRB2_complex	1
SHP2_GRB2_complex	1
Sirt1_M1_macrophage___nucleus	0

Sirt1_M2_macrophage__cytoplasm	0
SKIP	1
smad1_phosphorylated	0
SMAD2_phosphorylated_M1_macrophage___Cytoplasm	0
SMAD2_phosphorylated_M2_macrophage__cytoplasm	0
SMAD2_SARA_complex_M1_macrophage___Cytoplasm	0
SMAD2_SARA_complex_M2_macrophage__cytoplasm	0
SMAD2_SMAD4_complex_M1_macrophage___nucleus	0
SMAD2_SMAD4_complex_M2_macrophage_nucleus	0
SMAD4_M1_macrophage___Cytoplasm	0
SMAD4_M2_macrophage__cytoplasm	0
smad4_smad1_complex	0
SMAD7_Fibroblast___Cytoplasm	1
SMAD7_M1_macrophage___Cytoplasm	1
SMAD7_M2_macrophage__cytoplasm	1
snw1	1
SOCS3_rna	1
SOS1_Fibroblast___Cytoplasm	1
SOS1_M1_macrophage___Cytoplasm	1
SOS1_M2_macrophage__cytoplasm	1
SOS1_TH1___cytoplasm	1
Src_M1_macrophage___Cytoplasm	1
Src_M2_macrophage__cytoplasm	1
SRC_phosphorylated	1
Src_TH1___cytoplasm	1
sst	0
sst_sstr_complex	0
STAT1_Fibroblast___Cytoplasm	1
STAT1_Fibroblast___nucleus	1
STAT1_M1_macrophage___Cytoplasm	1
STAT1_STAT1_complex_M1_macrophage___nucleus	1
STAT1_STAT1_complex_TH1___nucleus	1
STAT1_STAT2_complex	1
STAT1_STAT2_IRF9_complex	1
STAT1_TH1___cytoplasm	1
STAT2_Fibroblast___Cytoplasm	1
STAT2_M1_macrophage___Cytoplasm	1
STAT3_Fibroblast___Cytoplasm	1
STAT3_Fibroblast___nucleus	1
STAT3_M1_macrophage___Cytoplasm	1
STAT3_M2_macrophage__cytoplasm	1
STAT3_STAT3_complex_M1_macrophage___nucleus	1
STAT3_STAT3_complex_M2_macrophage_nucleus	1
STAT4_M1_macrophage___Cytoplasm	1
STAT4_STAT4_complex_M1_macrophage___nucleus	1
STAT4_STAT4_complex_TH1___nucleus	1

STAT4_TH1___cytoplasm	1
STAT5_CRKL_complex	1
STAT5_phosphorylated	1
STAT6_STAT6_complex	1
SYK_M1_macrophage___Cytoplasm	0
SYK_M2_macrophage__cytoplasm	0
Syk_phosphorylated	1
TAB1	1
TAB1_TAB2_complex	1
TAB1_TAB2_TAK1_complex	1
TAB1_TAB2_TRAF6_complex	1
TAB2_phosphorylated	1
TAK1	1
TAK1_phosphorylated	1
tBID	0
TBK1	1
TBK1_IKBKE_complex	1
tbx21	1
tbx21_phosphorylated	1
TCF_LEF	1
TCF_LEF	1
TCR_CD3_complex	1
TGFb1	1
TGFB1_Fibroblast___Extracellular_Space	1
TGFB1_Fibroblast___Extracellular_Space_active	1
TGFB1_M2_macrophage__extracellular_space	1
TGFB1_M2_macrophage__secreted_components	1
TGFB1_TGFBR1_complex	1
TGFB1_TH1___secreted_components	1
tgfb3	0
TGFBR1_TGFBR2_complex	1
TGFBR1_TGFBR2_TGFB1_complex	1
THBS1_rna	0
TICAM1	1
TICAM1_TICAM2_complex	1
TICAM2	1
TIRAP_MYD88_complex	1
TLR1_TLR2_biglycan_complex	1
TLR1_TLR2_complex	1
TLR2_TLR6_biglycan_complex	1
TLR2_TLR6_complex	1
TLR3_dsRNA_complex	1
TLR4_Md2_CD14_fibrinogen_complex	0
TLR5_Fibroblast__Cytoplasmic_membrane_up	1
TLR5_M1_macrophage__Cytoplasmic_membrane_up	0
TLR7_TLR8_ssRNA_complex	1

TLR9_DNA_complex	1
TNF_Fibroblast___Extracellular_Space	1
TNF_Fibroblast___secreted_components	1
TNF_M1_macrophage___Extracellular_Space	1
TNF_M1_macrophage___Secreted_components	1
TNF_TH1___extracellular_space	1
TNF_TNFRSF1A_B_complex	1
TNF_TNFRSF1A_complex	1
TNF_TNFRSF1B_complex	1
TNFA	1
TNFA_rna	1
TNFAIP3_rna_M1_macrophage___nucleus	1
TNFAIP3_rna_M2_macrophage_nucleus	1
TNFRSF10A_rna	1
TNFRSF10B_rna	1
TNFSF11_Fibroblast___Extracellular_Space	1
TNFSF11_Fibroblast___secreted_components	1
TNFSF11_M1_macrophage___Extracellular_Space	1
TNFSF11_TH1___secreted_components	1
TNFSF11_TNFRSF11_complex	1
TNFSF4	1
TNFSF4_TNFRSF4_complex	1
TP53_phosphorylated	1
TP73_phosphorylated	0
TPL2_M1_macrophage___Cytoplasm	1
TPL2_M2_macrophage__cytoplasm	1
TRADD_Fibroblast___Cytoplasm	1
TRADD_M1_macrophage___Cytoplasm	1
TRADD_TRAF2_RIP1_complex	1
TRAF1_rna_Fibroblast___nucleus	1
TRAF1_rna_M1_macrophage___nucleus	1
TRAF1_rna_M2_macrophage_nucleus	1
TRAF1_TRAF2_TRAF3_complex	1
TRAF2_RIP1_TRADD_TAK1_TAB1_TAB2_complex	1
TRAF2_TRAF5_complex	1
TRAF2_TRAF5_complex	1
TRAF2_TRAF5_TRAF6_complex_TH1___cytoplasm	1
TRAF2_TRAF5_TRAF6_complex_TH1___cytoplasm_active	1
TRAF2_TRAF6_complex	1
TRAF3_Fibroblast___Cytoplasm	1
TRAF3_M1_macrophage___Cytoplasm	0
TRAF3_TH1___cytoplasm	1
TRAF3_TRAF6_complex	1
TRAF3IP2_phosphorylated_Fibroblast___Cytoplasm	1
TRAF3IP2_phosphorylated_M2_macrophage__cytoplasm	1
TRAF6_ECSIT_MEKK1_TAB1_TAB2_TAK1_complex	0

TRAF6_Fibroblast___Cytoplasm	1
TRAF6_IRAK1_IRAK4_complex	0
TRAF6_M1_macrophage___Cytoplasm	0
TRAF6_phosphorylated_Fibroblast___Cytoplasm	1
TRAF6_phosphorylated_TH1___cytoplasm	1
TRAF6_TAB1_TAB2_TAK1_complex	0
TRAF6_ubiquitinated_M1_macrophage___Cytoplasm	0
TRAF6_ubiquitinated_M2_macrophage__cytoplasm	0
TRAM1	0
TRAM1_TRIF_complex	0
TRIF	1
TSC2_phosphorylated	0
TSG6	1
TXK	1
TYK2	1
UEV1A_UBC13_complex	1
VAV1	0
VAV1_2_3	1
VCAM1_Fibroblast__Cytoplasmic_membrane_down	1
VCAM1_ITGA4_ITGB7_complex	1
VCAM1_TH1___cytoplasmic_membrane_down	1
vcan	1
VEGFa_Fibroblast___Extracellular_Space	1
VEGFa_Fibroblast___secreted_components	1
VEGFa_M2_macrophage__extracellular_space	1
VEGFa_M2_macrophage__secreted_components	1
VEGFA_rna	1
Vegfa_rna_M1_macrophage___nucleus	1
Vegfa_rna_M2_macrophage_nucleus	1
VEGFA_VEGFR_complex	1
Vegfb_M2_macrophage__extracellular_space	0
Vegfb_M2_macrophage__secreted_components	0
vegfc	1
Vegfc_M2_macrophage__extracellular_space	1
Vegfc_M2_macrophage__secreted_components	1
Vegfc_rna	1
vegfc_vegfr3_complex	1
VegfR1	1
VegfR1_Vegfa_complex	1
VegfR1_vegfb_complex	0
VegfR2_vegfc_complex	1
WNT_FRIZZLED_complex	1
WNT5A	1
WNT5B	1
WNT5B__rna	1
WNT5B_FZD1_LRP5_complex	1

WNT5B_gene	1
XIAP_M1_macrophage___Cytoplasm	1
XIAP_M2_macrophage__cytoplasm	1
YAP1_phosphorylated	1
YY1	1
ZAP70	0
ZC3H12A	0

Supplementary Table 10. Therapeutic drug targets in the RA multicellular model

Therapeutic Targets					
PDE4B	EGFR	CTSK	BIRC2	MAPK3	KRAS
SRC	JAK1	FAS	XIAP	ITGB1	IL7R
MAP3K1	CSF2	ACVR2B	MAP4K4	HIF1A	LAG3
MAP3K2	IL15	FGFR4	RIPK1	ITGA5	CD28
IKBKB	DKK1	CHUK	PTK2	ADAMTS4	TGFB1
GSK3B	PDCD1	IRAK4	PPIA	CXCL8	LRP5
CCNB1	IL2	BCL2	TLR2	MYC	NOTCH1
SIRT1	IL9	CCR5	TLR4	PTPRC	LIFR
MAPK1	PPARG	C5AR1	MIF	STAT3	CSF2RB
RPS6KB1	IL1A	PRKACA	CDC25B	MAP2K2	CTNNB1
MAPK8	ICAM1	TBK1	FGF1	RAC1	SEMA4D
MAPK14	BTK	MAP3K5	PLA2G2A	IL1B	ICOS
LCK	VEGFA	GALR2	ITGAL	CCL2	IL1R1
AKT1	PTPN11	IRAK1	PRKCQ	RHOA	IL4
PTPN6	MTOR	LYN	IL6R	IL17A	ITGA1
PTGS2	CXCR4	TYK2	IL6ST	STAT1	TNFRSF4
TLR9	IL23R	MAPK9	TNF	IFNG	ICOSLG
TLR8	IL4R	CXCR2	CXCL10	CD40LG	GAS6
TLR7	SYK	ZAP70	SERPINE1	CXCL12	VEGFC
MMP1	NRAS	PDGFRB	TP53	TNFSF11	BMP6
MMP14	CXCR3	MAP3K8	CASP9	IL6	NFKBIA
MMP3	RAF1	MERTK	DUSP1	CCL5	BAX
MMP9	JAK3	MAPKAPK2	JUN	HSPA5	IFNAR2
MMP13	F2R	TGFBR2	NFKB1	CD86	SMAD7
HCK	TGFBR1	AKT2	NFKB2	CD80	ACVR2A
MCL1	MAP3K7	CASP1	RELA	KLF4	IL18
BRAF	CCR2	CASP7	RELB	CCL20	FN1
ITGB7	INHBA	INHBB	PRLR	CSF1	IL17RA
THBS1	CSF1R	CASP8	EPHB2	CXCR1	CREB1
MIR221	CD40	NOS2	PTK2B	ITGB2	
MET	ITGA4	NLRP3	CCND1	IL17RC	
MDM2	ACKR3	IGF1R	ITGAV	CAV1	
JAK2	CASP3	PRKCD	MAP2K1	TBX21	
